# Depression and daytime dysfunction centralize the fatigue–sleep cascade in island firefighters: a symptom network and Bayesian DAG study

**DOI:** 10.3389/fpsyt.2025.1663957

**Published:** 2025-10-29

**Authors:** Yudan Liu, Zhihong Li, Qiong Xiang, Xue Zhang, Runhua Bai, Chenjing Sun, Jianguo Liu

**Affiliations:** ^1^ Department of Neurology, The Sixth Medical Center, Chinese People’s Liberation Army of China (PLA) General Hospital, Beijing, China; ^2^ School of Medicine, South China University of Technology, Guangzhou, China

**Keywords:** sleep disturbance, fatigue, psychological distress, psychological resilience, shiftwork, symptom network analysis, Bayesian Directed Acyclic Graph

## Abstract

**Background:**

Sleep disturbances, fatigue, and psychological distress are prevalent among island-based firefighters, a high-risk occupational group. However, the interactions and mechanisms underlying these factors remain unclear. This study investigated relationships among fatigue, sleep disturbances, psychological distress, and psychological resilience using symptom network analysis and exploratory Bayesian Directed Acyclic Graph (DAG) modeling.

**Methods:**

We surveyed 570 male island-based firefighters in China (cross-sectional). The PSQI, FSS, SCL-90, and CD-RISC were administered. Variables were residualized for demographic/behavioral covariates and z-standardized. We estimated an EBICglasso Gaussian Graphical Model (γ = 0.50) to quantify centrality (Strength, expected influence) and predictability (R²). Robustness was assessed via γ = 0.25–0.75 sensitivity, bootstrapping, and Network Comparison Tests across sleep status (sleep-disturbed [SD] vs sleep-normal [SN]) and work type (shift work [SW] vs non-shift [NS]). Exploratory Bayesian DAG modeling was conducted in SD using parallel Tabu/Hill-Climbing with BIC scoring and bootstrapped aggregation to derive a CPDAG.

**Results:**

Sleep disturbance prevalence was 46.0% (262/570). In the full network, depression (S4) and daytime dysfunction (P7) were among the most central nodes (EI = 1.938 and 1.613), and the fatigue total (F0) showed the highest predictability (R² = 0.176). In SD, hostility (S6, EI = 1.913) and anxiety (S5, EI = 1.462) emerged as potential affective hubs; tenacity (C1) was positioned upstream (Strength = 1.961; EI = −1.315) in relation to sleep and depression. Compared with SN, SD showed lower density and global strength (both P < 0.01). Between SW and NS, overall network structure differed (P = 0.014) whereas global strength did not (P = 0.694). Sensitivity analyses indicated high agreement of non-zero edges and minimal fluctuations in density/global strength across γ = 0.25–0.75. The DAG/CPDAG suggested a potential path from subjective sleep quality → fatigue → depression → hostility → somatization, with C1 potentially influencing sleep and depression; directionality warrants further longitudinal validation.

**Conclusion:**

Depression (S4) and daytime dysfunction (P7) may serve as key nodes linking sleep and affective processes; fatigue may relate to psychological distress via sleep; and tenacity (C1) could play an upstream protective role. Sleep status and shift work may reorganize network structure without necessarily altering global connectivity. Targeted interventions may consider subjective sleep perception and psychological resilience in island-based firefighters.

## 1 Introduction

Sleep disturbances have emerged as a pressing global public health challenge, affecting approximately 30% of adults worldwide (1). Such conditions not only elevate the risk of chronic illnesses but also impair neurocognitive functioning and increase the likelihood of accidents, thereby imposing substantial societal and healthcare burdens (2). Among high-risk occupational groups, island firefighters face compounded stressors including extreme work demands, prolonged geographic isolation, and repeated trauma exposure, rendering them particularly vulnerable to sleep disturbances. Meta-analytic evidence suggests that the comorbidity rate of sleep disorders among firefighters is 30.49% (95% CI: 25.90–35.06), while the prevalence of poor sleep quality reaches 51.43% (95% CI: 42.76–60.10) (3). Existing studies highlight shift work, psychological distress, and trauma exposure as major contributors to sleep disruption, all of which synergistically elevate the risk of operational errors, chronic fatigue, and burnout (4).

An increasing body of evidence supports a bidirectional relationship between sleep disturbance and psychological distress: symptoms of anxiety and depression interfere with sleep, while poor sleep in turn exacerbates emotional dysregulation (5–9). This dynamic interplay is especially pronounced in high-stress professions. Shift work has been identified as a key disruptor of circadian rhythm homeostasis, and its exposure correlates positively with risks of insomnia, fatigue, and mood disorders (10–13). However, not all individuals exposed to these occupational stressors develop psychopathology, suggesting the presence of protective psychological resources. Resilience, conceptualized as a cross-context adaptive capacity encompassing adaptability, emotion regulation, and tenacity in goal pursuit, has been widely recognized as a buffer against stress-related mental health risks (14–17).

Despite these advances, two major gaps remain in the literature: (1) most studies adopt a variable-centered, main-effect approach, overlooking the dynamic symptom interconnections and maintenance mechanisms; (2) the moderating role of occupational context—particularly shift work—on symptom networks has not been systematically examined.

To address these gaps, this study adopted a network psychopathology framework grounded in emotion regulation theory and resilience mechanisms, aiming to explore the systemic associations among sleep disturbance, fatigue, psychological distress, and resilience in island firefighters (18, 19). We hypothesized that these variables would exhibit specific network patterns, with daytime dysfunction and emotional distress occupying central positions as bridge symptoms, and resilience exhibiting upstream protective connections. Contextual factors such as shift work and sleep status may further moderate these network structures.

To test these hypotheses, we employed two complementary analytic frameworks: symptom network analysis (SNA) and Bayesian-directed acyclic graph (DAG) modeling. SNA, rooted in graph theory, treats symptom dimensions as nodes and partial correlations as edges, quantifying node influence via strength and expected influence (EI), and node predictability via R² (20–25). DAG modeling, as an exploratory causal inference tool, identifies potential directional dependencies to generate testable hypotheses about underlying mechanisms.

Accordingly, this study aimed to: (1) construct a symptom network encompassing fatigue, sleep disturbance, psychological distress, and resilience among island firefighters; (2) compare global and centrality features across sleep status (sleep-disturbed vs. normal) and work schedule (shift vs. non-shift) subgroups; and (3) explore potential directional paths among these domains via DAG modeling within the sleep-disturbed subgroup. The novelty of this study lies in integrating the complementary strengths of SNA and DAG, applying a dual-dimensional grouping strategy (sleep × shift) to systematically characterize the mental-sleep network of a high-risk occupational cohort, thereby providing mechanistic insights and testable foundations for precision interventions.

## 2 Methods

### 2.1 Participants

This study employed a cross-sectional design and was conducted in July 2023. Stratified cluster random sampling was used to recruit firefighters stationed on islands. Stratification was based on geographic location and administrative jurisdiction. Within each stratum, entire fire stations were randomly selected as sampling units, and on-duty personnel were sampled according to the daily shift schedule.

Inclusion criteria were: (1) employment duration of at least one month; (2) ability to complete the survey while on duty; and (3) provision of written informed consent.

Covariates were collected to control for potential confounding, including sociodemographic (age, gender, marital status, educational level, years of service, only-child status) and behavioral variables (smoking, tea consumption, caffeine intake, other stimulant use). Note: Data on alcohol use were not collected due to an on-duty alcohol prohibition policy.

A total of 610 questionnaires were distributed, and 609 were returned (response rate: 99.84%). After excluding invalid responses due to missing key variables, logical inconsistencies, patterned responses, or refusal, 578 valid questionnaires were retained (validity rate: 94.90%).

Given the limited number of female participants (n = 8) and the absence of sex-stratified hypotheses, only male participants (n = 570) were included in the final analysis to avoid estimation instability.

In the work schedule comparison, only fixed day shift (NS) and fixed rotating shift (SW) personnel were included; those with irregular or on-call duties were excluded to reduce exposure misclassification.

### 2.2 Measurement instruments

#### 2.2.1 Sleep quality

The Chinese version of the Pittsburgh Sleep Quality Index (PSQI) (26) was used to assess subjective sleep quality over the past month. It contains 19 items across seven components: subjective sleep quality (P1), sleep latency (P2), sleep duration (P3), habitual sleep efficiency (P4), sleep disturbances (P5), use of sleep medication (P6), and daytime dysfunction (P7). Each component is scored from 0 to 3, with a total score ranging from 0 to 21; higher scores indicate poorer sleep. A cutoff score of >7 was used to define clinically significant sleep disturbance, based on prior validation studies in Chinese adult populations demonstrating good internal consistency and test-retest reliability (e.g., α ≈ 0.84, ICC ≈ 0.81) (27). In this study, Cronbach’s α was 0.85.

#### 2.2.2 Fatigue severity

Fatigue was measured using the Fatigue Severity Scale (FSS) (28), which assesses subjective fatigue over the past week. The scale contains 9 items rated on a 7-point Likert scale (1 = “strongly disagree” to 7 = “strongly agree”), yielding a total score from 9 to 63. Higher scores indicate more severe fatigue. A total score ≥36 or average score ≥4 was used to indicate high fatigue, as commonly applied in clinical and occupational studies. The Chinese version has demonstrated good internal consistency (α ≈ 0.93) and acceptable construct validity (29). In this study, Cronbach’s α was 0.92.

#### 2.2.3 Psychological distress

Psychological distress was assessed using the Symptom Checklist-90 (SCL-90) (30), a 90-item self-report measure with 10 dimensions: somatization (S1), obsessive-compulsiveness (S2), interpersonal sensitivity (S3), depression (S4), anxiety (S5), hostility (S6), phobic anxiety (S7), paranoid ideation (S8), psychoticism (S9), and additional symptoms (S10). Each item is scored on a 5-point Likert scale (1 = “not at all” to 5 = “extremely”). A total score ≥160 or any subscale mean ≥2 indicated elevated psychological distress. The Chinese version has been validated in general populations with high internal consistency (31). In this study, Cronbach’s α = 0.93.

#### 2.2.4 Psychological resilience

Psychological resilience was measured using the 25-item Connor-Davidson Resilience Scale (CD-RISC) (32), which includes three dimensions: tenacity (C1), strength (C2), and optimism/control (C3). Items are rated on a 5-point Likert scale (1 = “not true at all” to 5 = “true nearly all the time”), with total scores ranging from 25 to 125. Higher scores indicate greater resilience. The Chinese version has demonstrated strong internal consistency (α ≈ 0.91) and structural validity across adolescent and adult samples (33). In this study, Cronbach’s α was 0.93.

### 2.3 Common-method bias assessment

To assess potential common-method bias, we conducted Harman’s single-factor test. Eleven factors with eigenvalues greater than 1 were extracted, and the first factor accounted for only 26.51% of the total variance—well below the conventional 40% threshold, indicating no significant common-method bias.

### 2.4 Statistical analyses

All analyses and visualizations were conducted using R (version 4.4.2). All variables were z-standardized prior to network estimation.

#### 2.4.1 Descriptive statistics and correlation analysis

Descriptive statistics and group comparisons (SD vs. SN; SW vs. NS) were performed using the compareGroups package. To reduce confounding effects, we first regressed all network variables on covariates—including age, marital status, education, years of service, only-child status, smoking, tea, caffeine, and other stimulant use—and extracted standardized residuals. All network and DAG analyses were based on these residualized scores. Spearman correlations were computed to estimate bivariate associations among residualized variables and visualized using the corrplot package.

#### 2.4.2 Network estimation and visualization

Gaussian Graphical Models (GGMs) were estimated to identify conditional dependencies among symptom dimensions. The EBICglasso method (γ = 0.50) was applied, combining graphical LASSO regularization with the Extended Bayesian Information Criterion to balance sparsity and model fit. All input variables were z-standardized residuals obtained after covariate adjustment. To evaluate model robustness, we conducted two sensitivity analyses: (1) altering γ to 0.25 and 0.75, and (2) replacing the Spearman correlation matrix with a mixed-type correlation matrix estimated via cor_auto, which is more suitable for ordinal data. Twenty-one nodes were included in the network, covering the 7 PSQI components, 10 SCL-90 subscales, 3 CD-RISC dimensions, and the FSS total score. We chose to model at the domain/factor level rather than the item level to improve network interpretability and ensure stability in subgroup analyses, while acknowledging that this approach may mask item-level heterogeneity. Networks were visualized using the Fruchterman–Reingold layout. Red solid edges represent positive partial correlations; blue dashed edges represent negative ones; edge thickness reflects absolute edge weights.

#### 2.4.3 Centrality and predictability metrics

To identify key nodes within the symptom network, two centrality indices were calculated: (1) Strength – the sum of the absolute weights of all edges connected to a node, reflecting overall connectedness; (2) Expected Influence (EI) – the algebraic sum of edge weights (considering sign), which captures potential activation or inhibition effects. Additionally, predictability (R²) for each node was computed by regressing each variable on its directly connected neighbors, indicating how much of a node’s variance is explained by its adjacent nodes. All computations were performed using the mgm package.

#### 2.4.4 Network stability and accuracy

Stability and accuracy of the estimated network were assessed using the bootnet package.

Nonparametric bootstrapping (5,000 resamples) was used to generate 95% confidence intervals (CIs) for edge weights, indicating estimation precision. Centrality stability was evaluated via case-dropping bootstraps: random subsets ranging from 10% to 90% of the sample were removed, and the centrality estimates recalculated across 5,000 iterations. The resulting Correlation Stability (CS) coefficient was computed based on Pearson correlations between centrality estimates from subsets and those from the full sample, quantifying the robustness of centrality indices to sampling variation. A CS coefficient > 0.25 was considered acceptable; values > 0.50 were interpreted as indicating good stability.

#### 2.4.5 Network comparison tests

To compare network properties across subgroups, the NetworkComparisonTest (NCT) package was used to test for: (1) global strength differences (sum of all absolute edge weights); (2) overall network structure invariance; (3) individual edge differences. All tests were conducted using 5,000 permutations. Invariance test statistic (M) and global strength statistic (S) were used to evaluate differences. When global strength was not significantly different, we reported descriptive trends only.

#### 2.4.6 Bayesian DAG modeling

In the sleep-disturbed (SD) subgroup, exploratory Bayesian Directed Acyclic Graphs (DAGs) were constructed using the bnlearn package under the assumption of acyclicity (i.e., no feedback loops) (34). Both Tabu and Hill-Climbing (HC) structure learning algorithms were used in parallel, with the Bayesian Information Criterion (BIC) as the scoring metric. Bootstrap resampling (5,000 iterations) was used to estimate arc strength (i.e., edge frequency across bootstrap samples) and directional probabilities. The main analysis retained arcs with strength ≥ 0.50 and direction ≥ 0.80. Arcs with strength ≥ 0.20 were reported as sensitivity-level connections. We also generated a Completed Partially Directed Acyclic Graph (CPDAG) to reflect undirected or uncertain-direction arcs. Concordance between Tabu and HC algorithms was examined to evaluate model robustness. Importantly, DAGs based on cross-sectional data are exploratory in nature; inferred directional relationships are hypothesis-generating only and should be validated via longitudinal or interventional designs.

## 3 Results

### 3.1 Sample characteristics and group differences

**Figure 1:**
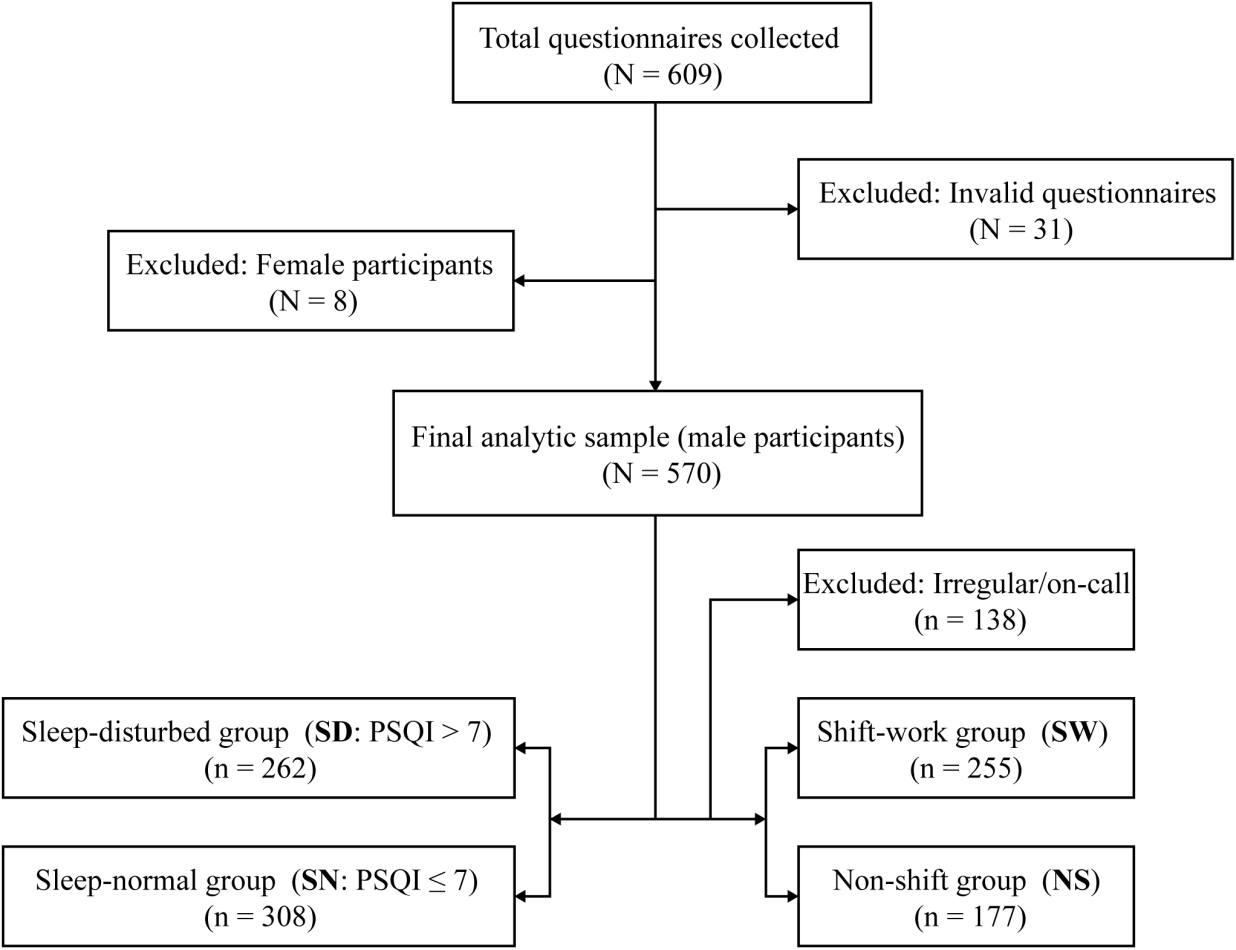
Flowchart of participant selection and sample allocation. A total of 609 questionnaires were collected. After excluding invalid questionnaires (n=31) and female respondents (n=8), 570 male participants were included in the full analytic sample. Participants were classified by sleep status into sleep-disturbed (SD; PSQI>7; n=262) and sleep-normal (SN; PSQI ≤ 7; n=308). For the shift-status analyses, individuals with irregular/on-call schedules were excluded (n=138), yielding 432 participants: shift-work (SW; n=255) and non-shift (NS; n=177). PSQI, Pittsburgh Sleep Quality Index; SD, sleep-disturbed; SN, sleep-normal; SW, shift-work; NS, non-shift.

A total of 570 male island firefighters were included in the final analysis (**Figure 1**). Based on the PSQI cutoff of >7, 262 participants (46.0%) were classified as sleep-disturbed (SD), and 308 (54.0%) as sleep-normal (SN). Regarding work schedule, 255 individuals (44.7%) were assigned to the shift-work group (SW), and 177 (31.1%) to the non-shift group (NS); an additional 138 participants (24.2%) were excluded from shift status comparisons due to irregular/on-call work patterns.

Baseline characteristics (**Supplementary Table S1**) revealed significant differences (P < 0.05) between the SD and SN groups in age, years of service, marital status, work schedule distribution (SW/NS), and lifestyle factors (smoking, tea drinking, caffeine intake, and other stimulant use). Similarly, the SW and NS groups differed significantly in coffee, tea, and other stimulant consumption (**Supplementary Table S2**), supporting the need to residualize all covariates in subsequent analyses.

Group-level symptom comparisons (**Supplementary Tables S3** and **S4**) indicated that the SD group had significantly higher scores across all PSQI sleep components, most SCL-90 distress dimensions (except phobic anxiety and psychoticism), and fatigue (F0) (all P < 0.001), while scoring lower on all CD-RISC resilience components (P < 0.001). The SW group also scored significantly higher than the NS group on multiple sleep components (P1, P2, P3, P5, P7), psychological distress dimensions (S1–S6, S8, S10), and fatigue (F0), with significantly lower resilience scores on C1 and C2.

### 3.2 Correlations between symptom dimensions

**Figure 2:**
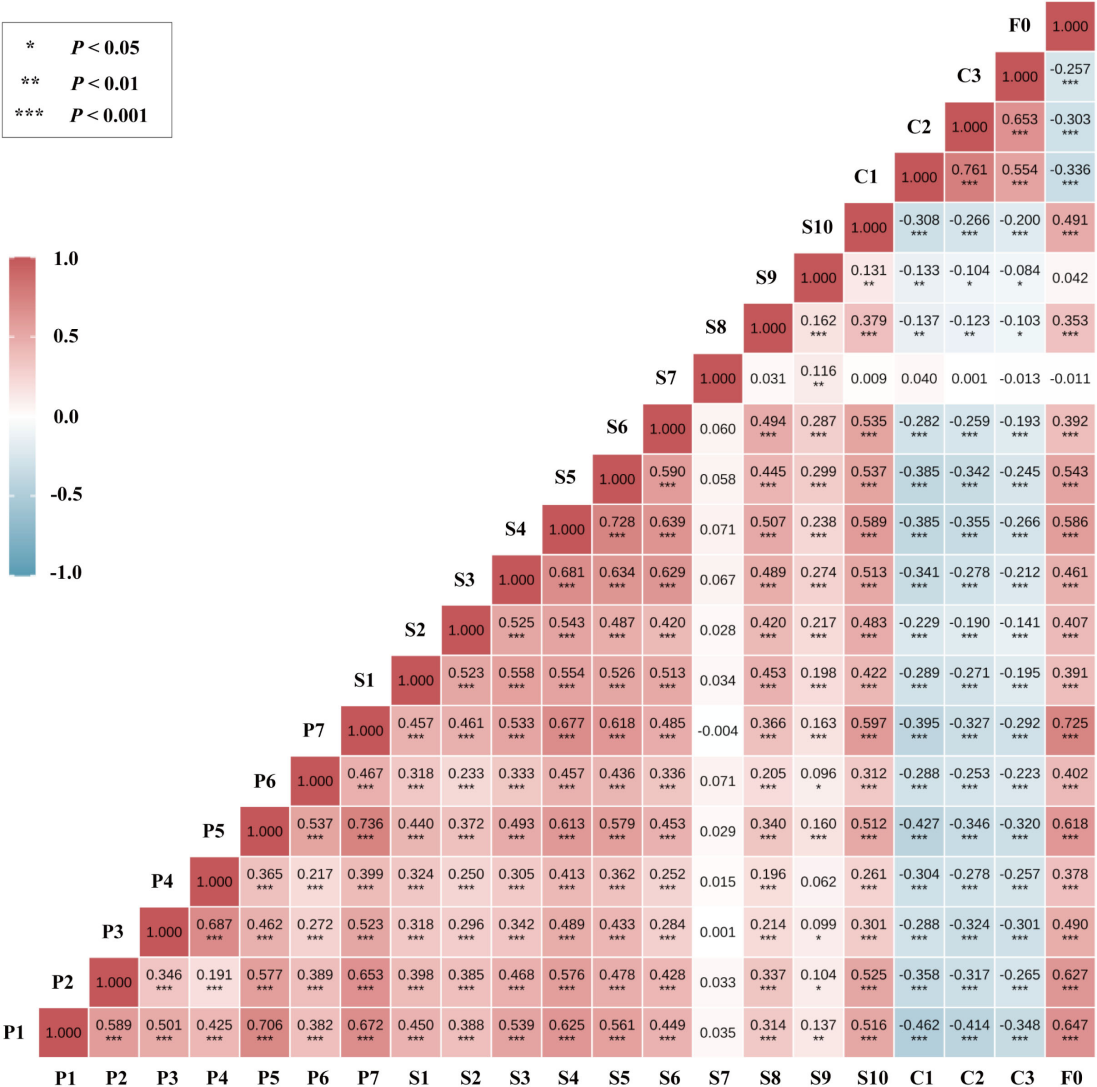
Variable classification and subgrouping strategy for network analysis. Variables were Z-standardized; correlations were computed on covariate-residualized scores (see Methods for prespecified covariates). The color scale denotes r from −1 to 1 (blue = negative; red = positive). Asterisks indicate two-sided significance (*P < 0.05; **P < 0.01; ***P < 0.001). P1–P7, PSQI components; S1–S10, SCL-90 factors; C1–C3, CD-RISC dimensions; F0, FSS total score.

After residualizing for covariates, the Spearman correlation matrix (**Figure 2**) revealed the strongest associations between fatigue (F0) and sleep-related dimensions: F0–P7 (r = 0.725), F0–P1 (r = 0.647), and F0–P2 (r = 0.627), all with P < 0.001.

Significant correlations were also observed between emotional symptoms and sleep dimensions, particularly S4–P7 (r = 0.677), S4–P1 (r = 0.625), and S5–P7 (r = 0.618), highlighting a strong affect–sleep connection in the network structure.

### 3.3 Overall network structure

#### 3.3.1 Visualization and centrality

**Figure 3:**
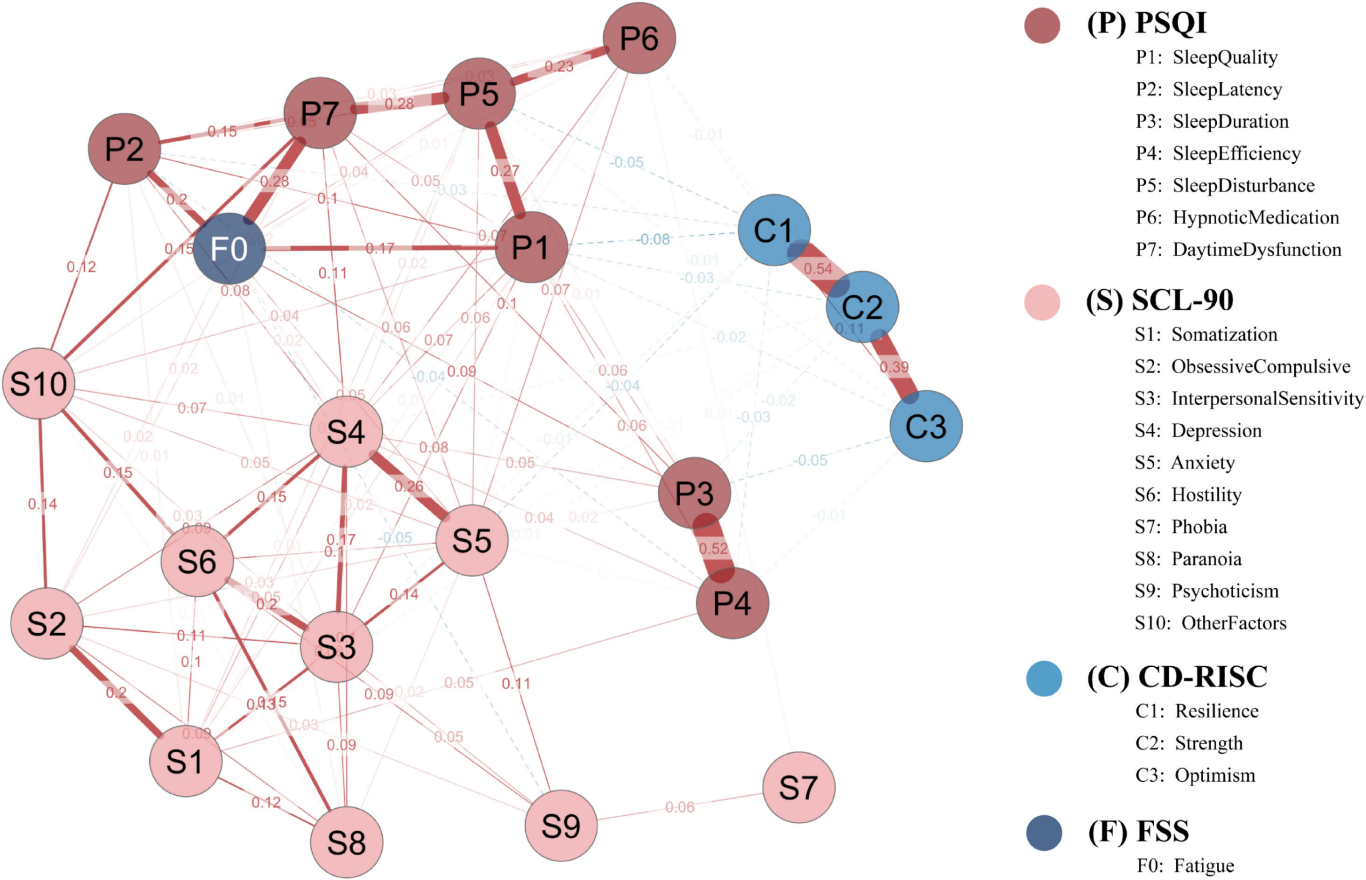
Network structure of sleep disturbance, fatigue, psychological distress, and resilience in the full sample. The network is a Gaussian graphical model estimated via EBICglasso (γ = 0.50) with Z-standardized variables. Node colors denote instrument domains (P = PSQI; S = SCL-90; C = CD-RISC; F = FSS). Solid red edges indicate positive partial correlations; dashed blue edges indicate negative partial correlations; edge thickness is proportional to the absolute edge weight. Node layout was determined by the Fruchterman–Reingold algorithm. P1–P7, PSQI components; S1–S10, SCL-90 subscales; C1–C3, CD-RISC factors; F0, FSS total score.

The overall symptom network comprised 21 nodes. Of the 210 possible edges, 104 remained after EBICglasso regularization (γ = 0.50), yielding a network density of 0.495 (**Figure 3**).

As shown in **Table 1**, depression (S4) and daytime dysfunction (P7) exhibited the highest centrality in both strength (1.741 and 1.324, respectively) and expected influence (EI = 1.938 and 1.613), suggesting their broad connectivity and high propagation potential.

**Table 1:** Centrality and predictability of symptoms in the full sample.

Node	Strength	Expected Influence	Predictability(R^2^)
P1	0.777	0.202	0.106
P2	-0.010	-0.132	0.152
P3	0.242	0.139	0.008
P4	-0.254	-0.455	0.011
P5	0.797	0.673	0.046
P6	-1.208	-0.982	0.142
P7	1.324	1.613	0.096
S1	-0.077	0.180	0.010
S2	-0.338	0.001	0.012
S3	0.653	0.873	0.058
S4	1.741	1.938	0.077
S5	0.851	0.846	0.104
S6	0.330	0.648	0.016
S7	-2.595	-2.190	0.151
S8	-0.846	-0.493	0.074
S9	-1.541	-1.462	0.001
S10	-0.213	0.122	0.147
C1	0.228	-1.124	0.002
C2	0.578	0.356	0.007
C3	-0.797	-1.134	0.015
F0	0.357	0.379	0.176

Strength and expected influence (EI) values were derived from EBICglasso-based partial correlation networks. Predictability (*R²*) indicates the proportion of variance in each node explained by its neighboring nodes. P, PSQI components (P1–P7); S, SCL-90 subscales (S1–S10); C, CD-RISC factors (C1–C3); F, FSS total score. Strength, absolute connectivity of a node; Expected Influence, signed connectivity; Predictability (R²), proportion of variance explained by neighboring nodes.

Anxiety (S5) also showed a notably high EI (0.846), indicating its possible regulatory role within the network.

Regarding predictability (R²), fatigue (F0) was the most predictable node (R² = 0.176), followed by sleep latency (P2, R² = 0.152) and phobic anxiety (S7, R² = 0.151), implying that these nodes were more easily explained by their neighbors.

#### 3.3.2 Network accuracy and stability

**Figure 4:**
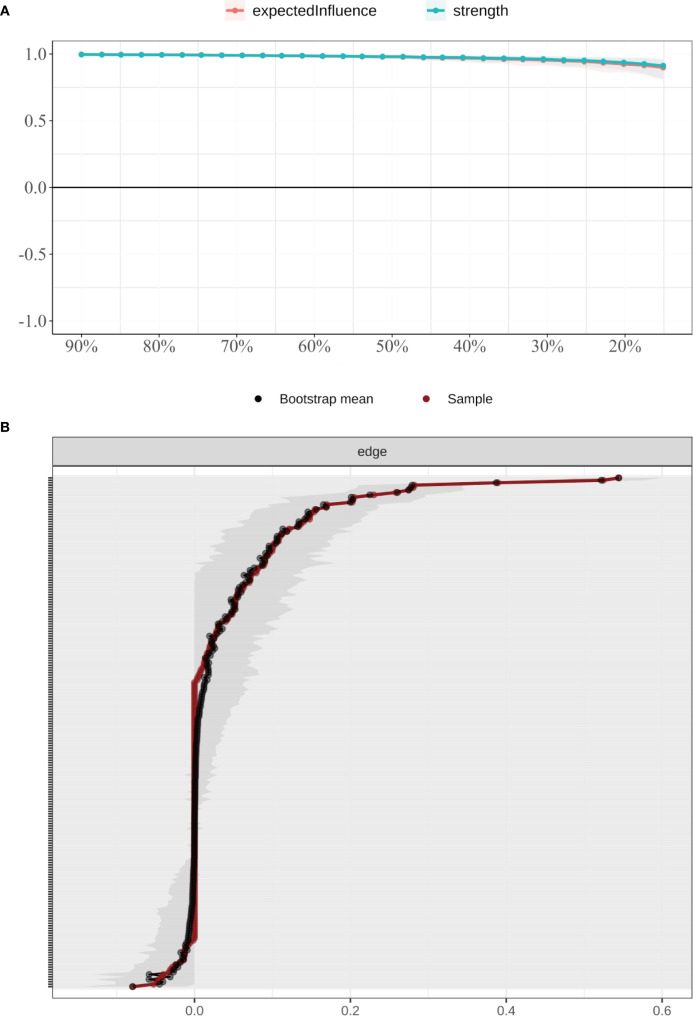
Stability of node centrality indices estimated by case-dropping bootstrap. **(A)** Case-dropping bootstrap stability of node centrality indices (strength and expected influence, EI). Curves show the average correlation between original centrality and centrality recomputed in subset samples across increasing case-dropping proportions. **(B)** Nonparametric bootstrap 95% confidence intervals for edge weights; black dots denote bootstrap means and the red line indicates the sample estimate, with narrower bands reflecting higher precision. EI, expected influence; P1–P7, PSQI components; S1–S10, SCL-90 subscales; C1–C3, CD-RISC factors; F0, FSS total score.

The accuracy and stability of the network were verified using case-dropping bootstrapping (**Figure 4A**). Even after removing up to 80% of cases, the Pearson correlations between the bootstrapped and original centrality metrics remained above 0.75. The correlation stability coefficient (CS) was 0.846, exceeding the recommended threshold of 0.50, suggesting robust centrality estimates. Bootstrap confidence intervals for edge weights (**Figure 4B**) revealed narrower intervals for stronger edges, while weaker edges showed greater uncertainty, indicating a need for cautious interpretation.

### 3.4 Subgroup network structures

#### 3.4.1 Network features by sleep status

**Figure 5:**
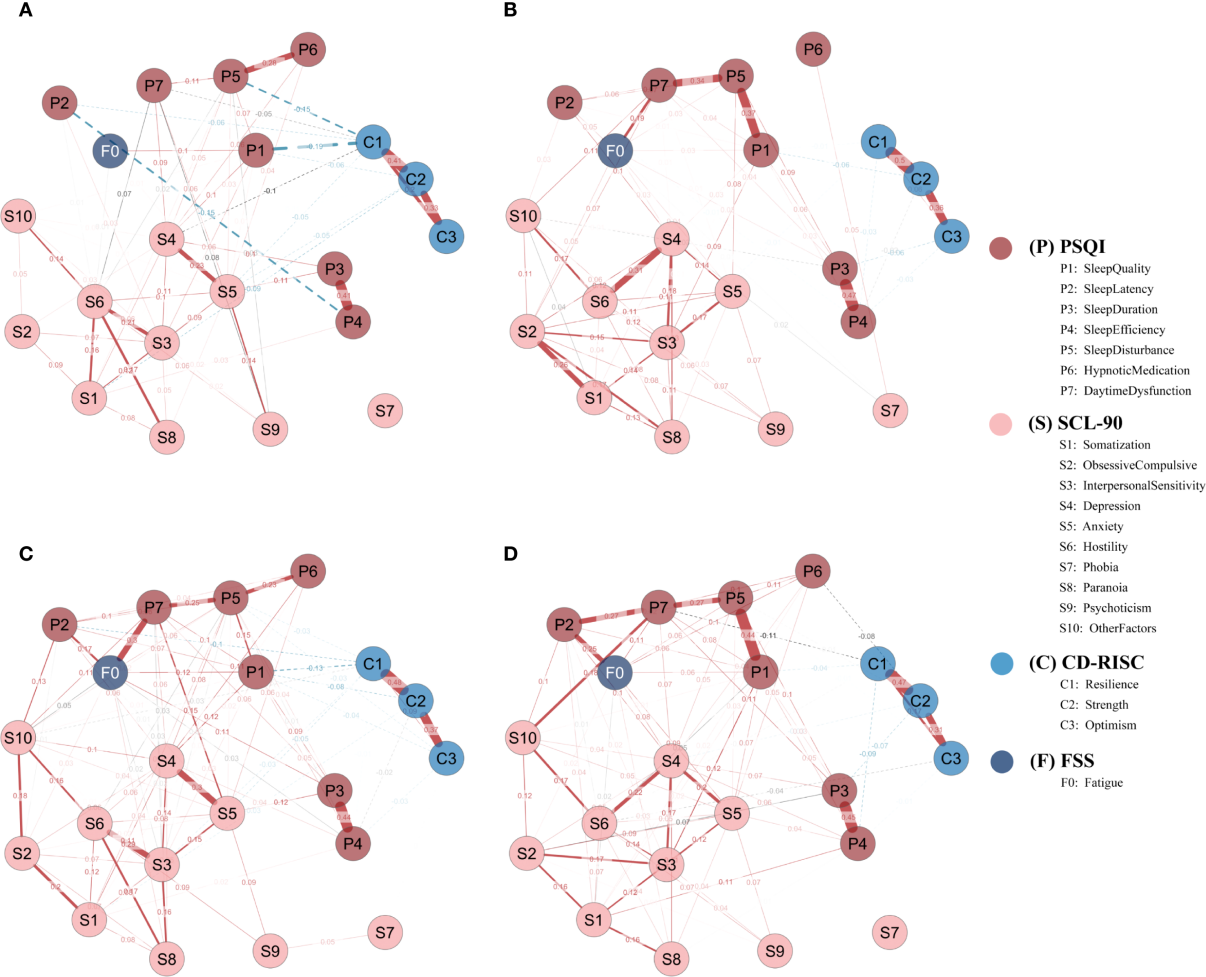
Subgroup network structures by sleep disorder and shift work status. **(A)** Sleep-disturbed (SD; n = 262); **(B)** Sleep-normal (SN; n = 308); **(C)** Shift-work (SW; n = 255); **(D)** Non-shift (NS; n = 177). Networks were estimated using EBICglasso (γ = 0.50) on Z-standardized variables, with covariates controlled as specified in the Methods (shift-status analyses exclude irregular/on-call schedules). Nodes represent symptom dimensions; node colors denote instrument domains (P = PSQI; S = SCL-90; C = CD-RISC; F = FSS). Solid red edges = positive partial correlations; dashed blue edges = negative partial correlations; edge thickness = absolute edge weight. Node layout = Fruchterman–Reingold algorithm. P1–P7, PSQI components; S1–S10, SCL-90 subscales; C1–C3, CD-RISC factors; F0, FSS total score; SD, sleep-disturbed; SN, sleep-normal; SW, shift-work; NS, non-shift.

Four subgroup networks (SD, SN, SW, NS) were constructed using EBICglasso (γ = 0.50) on residualized data, with consistent Fruchterman–Reingold layout for comparability (**Figure 5**).

In the SD group, the most central node by strength was resilience (C1 = 1.961), followed by anxiety (S5 = 1.369), hostility (S6 = 1.160), depression (S4 = 1.122), and interpersonal sensitivity (S3 = 0.626).

**Figure 6:**
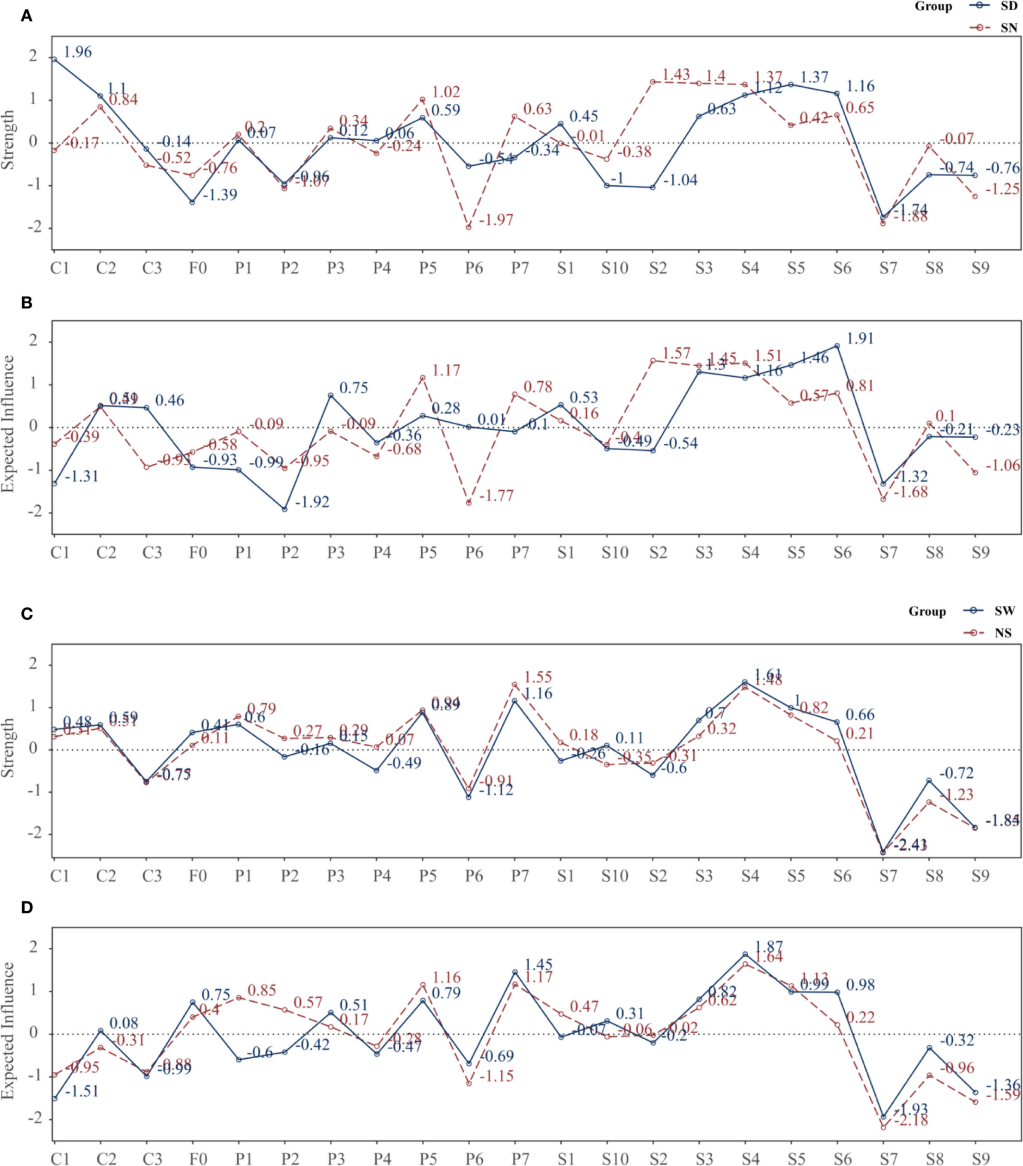
Strength and expected influence of nodes across subgroups. **(A)** Strength: SD vs SN; **(B)** Expected influence (EI): SD vs SN; **(C)** Strength: SW vs NS; **(D)** Expected influence (EI): SW vs NS. Centrality metrics were computed from subgroup-specific EBICglasso networks (γ = 0.50) estimated on Z-standardized variables, with covariates controlled as described in the Methods. Each point denotes a node’s standardized centrality score; higher values indicate greater influence within the network. Definitions: Strength, sum of absolute partial-correlation edge weights; EI, sum of signed edge weights. SD, sleep-disturbed; SN, sleep-normal; SW, shift-work; NS, non-shift; P1–P7, PSQI components; S1–S10, SCL-90 subscales; C1–C3, CD-RISC factors; F0, FSS total score.

In contrast, the SN group showed highest strength centrality for obsessive-compulsion (S2 = 1.431), interpersonal sensitivity (S3 = 1.398), and depression (S4 = 1.371), followed by sleep disturbance (P5 = 1.022) and hostility (S6 = 0.654) (**Figure 6A**).

Regarding EI, in the SD group, the most influential nodes were hostility (S6 = 1.913), anxiety (S5 = 1.462), and interpersonal sensitivity (S3 = 1.304), whereas C1 had a negative EI (–1.315), suggesting its involvement in a protective, non-propagating subnetwork.

In the SN group, top EI nodes included S2 (1.568), S4 (1.509), S3 (1.448), and P5 (1.167) (**Figure 6B**).

Predictability (R²) analyses revealed greater explanatory power in the SN group: anxiety (S5 = 0.340), paranoia (S8 = 0.334), psychoticism (S9 = 0.243), interpersonal sensitivity (S3 = 0.222), and hypnotic use (P6 = 0.186) were most predictable. In contrast, S6 and S7 had near-zero R² in the SD group.

These patterns suggest that the SN network was more structured around internalizing symptoms, while in the SD group, C1 played a central role, indicating a potential buffering function under conditions of sleep disturbance.

#### 3.4.2 Network features by work schedule

In the SW group, the nodes with highest strength were depression (S4 = 1.606) and daytime dysfunction (P7 = 1.161), followed by anxiety (S5 = 0.996), sleep disturbance (P5 = 0.886), and interpersonal sensitivity (S3 = 0.697).

In the NS group, P7 (1.546) and S4 (1.484) remained dominant, with stronger involvement of P5 (0.941), P1 (0.793), and S5 (0.823) (**Figure 6C**).

In terms of EI, the SW group was dominated by S4 (1.873), P7 (1.454), S5 (0.989), S6 (0.981), and S3 (0.818), whereas F0 (0.750) and P5 (0.788) also showed high propagation potential.

The NS group showed greater EI for sleep-related nodes such as S4 (1.642), P7 (1.167), P5 (1.155), S5 (1.131), and P1 (0.854) (**Figure 6D**).

Overall, the NS network was more centered on sleep pathways, while the SW network highlighted emotional and interpersonal propagation, suggesting greater emotional reactivity under shift work.

For predictability (R²), the SW group showed higher R² for hypnotics (P6 = 0.164), daytime dysfunction (P7 = 0.145), anxiety (S5 = 0.133), and phobic anxiety (S7 = 0.162).

In the NS group, the most predictable nodes were P2 (0.170), P6 (0.144), P5 (0.137), F0 (0.109), and P1 (0.118), indicating greater structural coherence along sleep pathways (**Table 2**).

**Table 2:** Comparison of node predictability (R²) between subgroups.

Node	SD (*n* = 262)	SN (*n* = 308)	SW (*n* = 255)	NS (*n* = 177)
P1	0.103	0.091	0.091	0.118
P2	0.020	0.114	0.068	0.170
P3	0.023	0.000	0.003	0.030
P4	0.012	0.008	0.010	0.019
P5	0.061	0.015	0.046	0.137
P6	0.057	0.186	0.164	0.144
P7	0.052	0.022	0.145	0.034
S1	0.032	0.222	0.023	0.001
S2	0.015	0.034	0.006	0.032
S3	0.105	0.141	0.082	0.083
S4	0.013	0.096	0.081	0.042
S5	0.013	0.340	0.133	0.032
S6	0.000	0.099	0.004	0.035
S7	0.000	0.108	0.162	0.000
S8	0.139	0.334	0.071	0.083
S9	0.142	0.243	0.004	0.034
S10	0.061	0.161	0.084	0.131
C1	0.008	0.029	0.000	0.002
C2	0.075	0.011	0.001	0.001
C3	0.211	0.029	0.019	0.016
F0	0.006	0.084	0.076	0.109

Predictability (R²) indicates the proportion of variance in each node explained by its neighboring nodes. R² was obtained from nodewise regressions in the EBICglasso Gaussian graphical model using residualized, z-standardized variables. SD, Sleep-disturbed (PSQI > 7); SN, Sleep-normal (PSQI ≤ 7); SW, Shift-work; NS, Non-shift; P, PSQI components (P1–P7); S, SCL-90 subscales (S1–S10); C, CD-RISC factors (C1–C3); F0, FSS total score. Values are reported to three decimals; values <0.0005 are shown as 0.000. Top-5 R² within each subgroup are bolded.

#### 3.4.3 Subgroup network accuracy and stability

**Figure 7:**
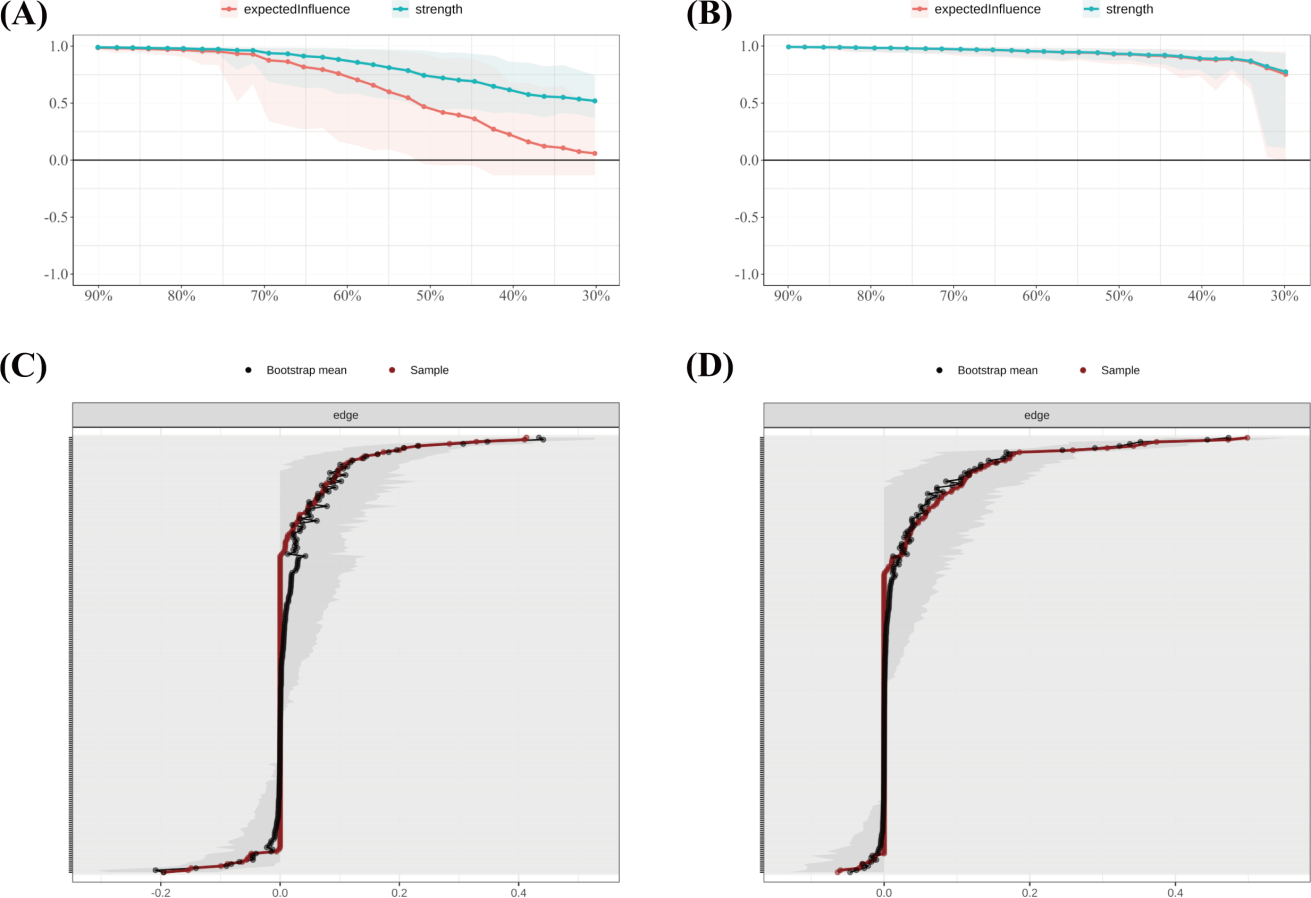
Stability of node centrality and edge-weight accuracy by sleep-status subgroup. **(A)** SD—case-dropping bootstrap stability of node centrality (strength & expected influence, EI); **(B)** SN—case-dropping bootstrap stability of node centrality (strength & EI); **(C)** SD—nonparametric bootstrap 95% confidence intervals for edge weights; SN—nonparametric bootstrap 95% confidence intervals for edge weights. Networks were estimated with EBICglasso (γ = 0.50) on Z-standardized variables, controlling covariates as described in the Methods. In (A–B), curves show the average correlation between original centrality and centrality recomputed in subset samples across case-dropping proportions; shaded ribbons = bootstrap bands (higher curves = better stability). In **(C, D)**, black dots = bootstrap means; red line = sample estimate; narrower bands = higher precision. Definitions: Strength = sum of absolute partial-correlation edge weights; EI = sum of signed edge weights. SD = sleep-disturbed; SN = sleep-normal; P1–P7 = PSQI components; S1–S10 = SCL-90 subscales; C1–C3 = CD-RISC factors; F0 = FSS total score.

**Figure 8:**
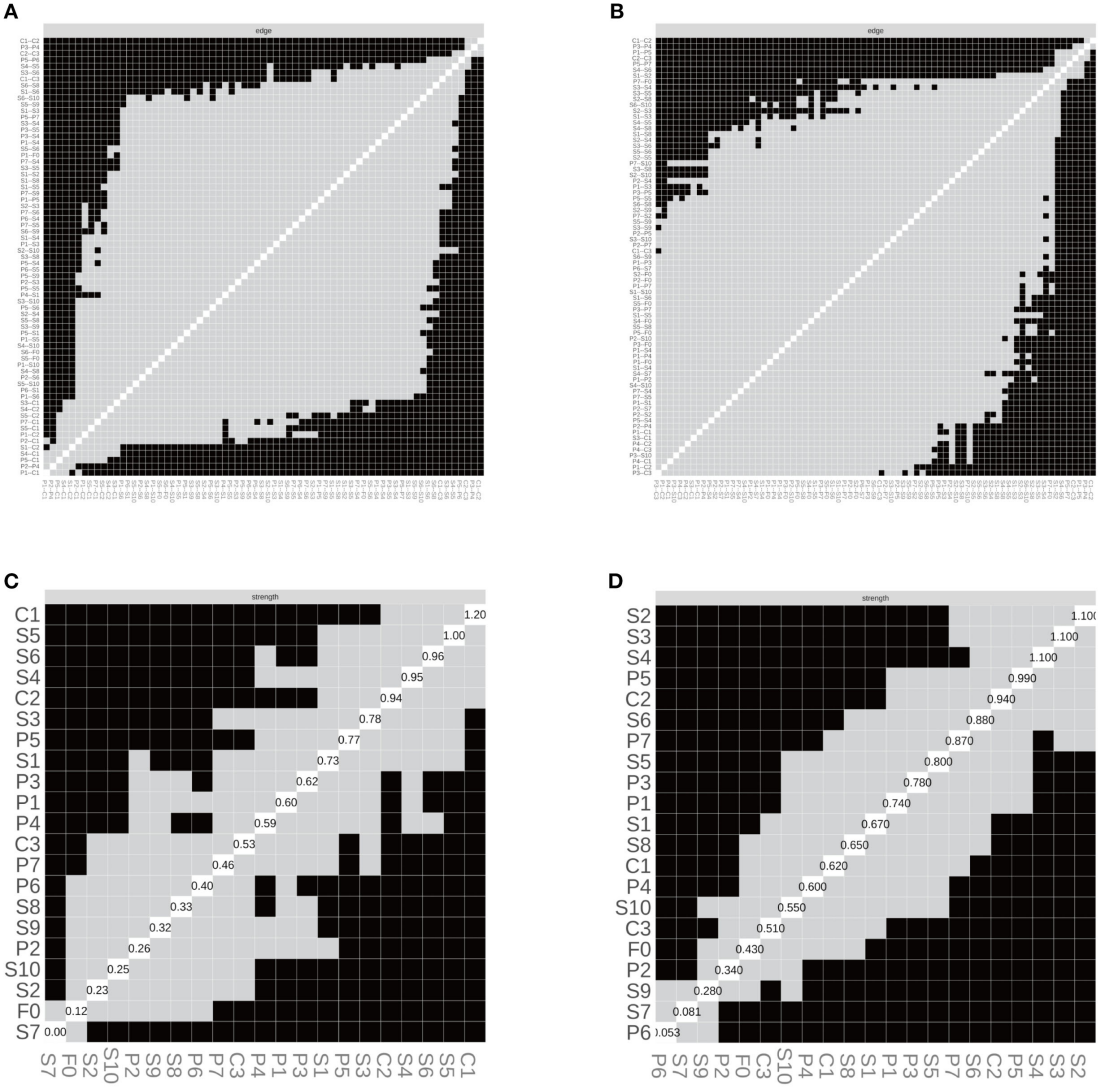
Edge structure and node strength by sleep-status subgroup. **(A)** SD—edge-selection matrix; **(B)** SN—edge-selection matrix; **(C)** SD—node-strength heatmap; **(D)** SN—node-strength heatmap. Networks were estimated with EBICglasso (γ = 0.50) on Z-standardized variables, with covariates controlled as described in the Methods. In (A–B), cells depict the presence of non-zero partial-correlation edges (gray = present, black = absent; diagonal = self). In **(C, D)**, shading reflects node-wise strength (lighter = larger), and the diagonal labels show each node’s standardized strength value (sum of absolute edge weights). SD = sleep-disturbed; SN = sleep-normal; P1–P7 = PSQI components; S1–S10 = SCL-90 subscales; C1–C3 = CD-RISC factors; F0 = FSS total score. Strength = sum of absolute partial-correlation edge weights.

**Figure 9:**
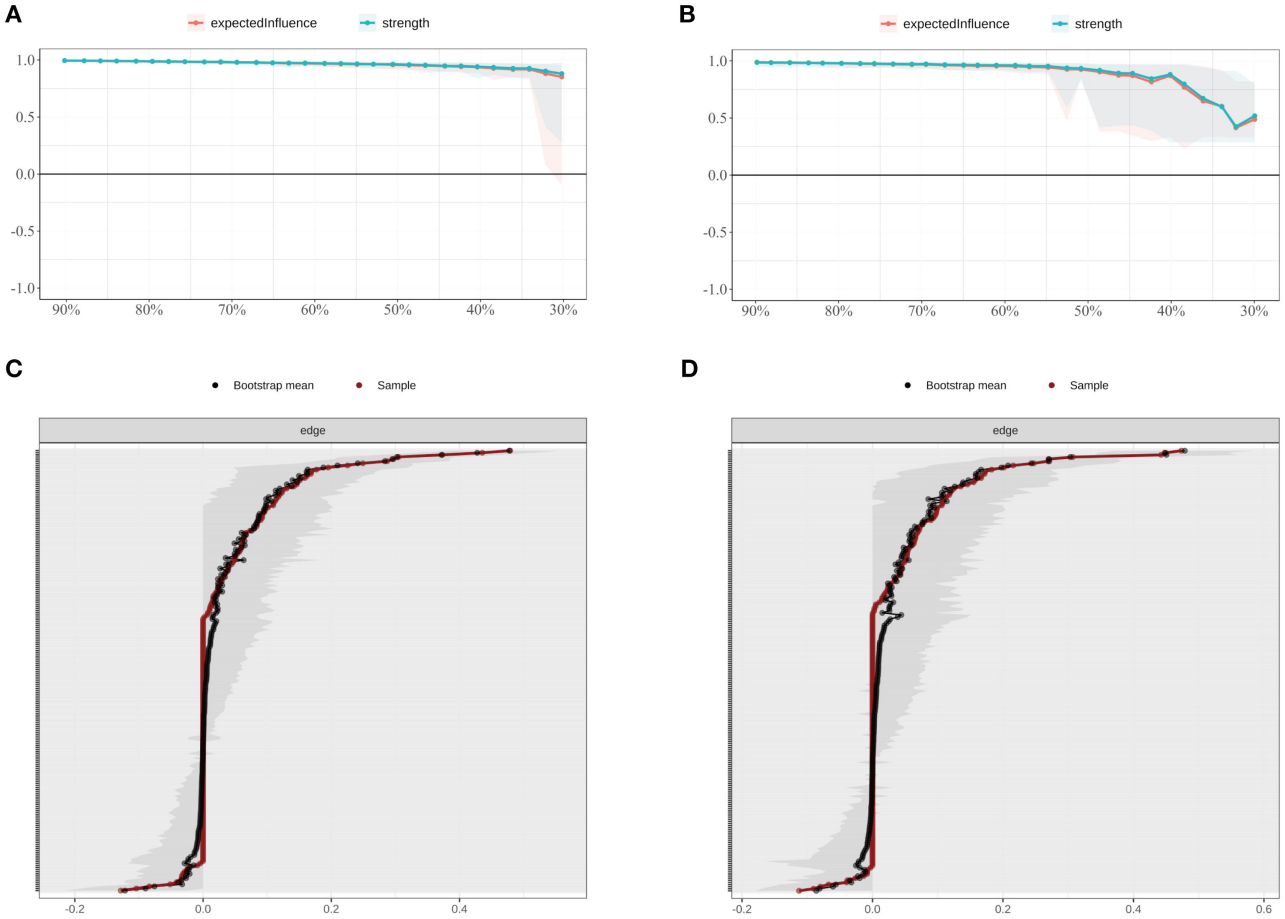
Stability of node centrality and edge-weight accuracy by shift-work subgroup. **(A)** SW—case-dropping bootstrap stability of node centrality (strength & EI); **(B)** NS—case-dropping bootstrap stability of node centrality (strength & EI); **(C)** SW—nonparametric bootstrap 95% confidence intervals for edge weights; **(D)** NS—nonparametric bootstrap 95% confidence intervals for edge weights. Networks were estimated with EBICglasso (γ = 0.50) on Z-standardized variables, with covariates controlled (shift-status analyses exclude irregular/on-call schedules). In (A–B), curves depict stability as the average correlation between original and subset centrality across case-dropping proportions; shaded ribbons = bootstrap bands. In (C–D), black dots = bootstrap means; red line = sample estimate; narrower bands = higher precision. Definitions: Strength = sum of absolute partial-correlation edge weights; EI = sum of signed edge weights. SW = shift-work; NS = non-shift; P1–P7 = PSQI components; S1–S10 = SCL-90 subscales; C1–C3 = CD-RISC factors; F0 = FSS total score.

**Figure 10:**
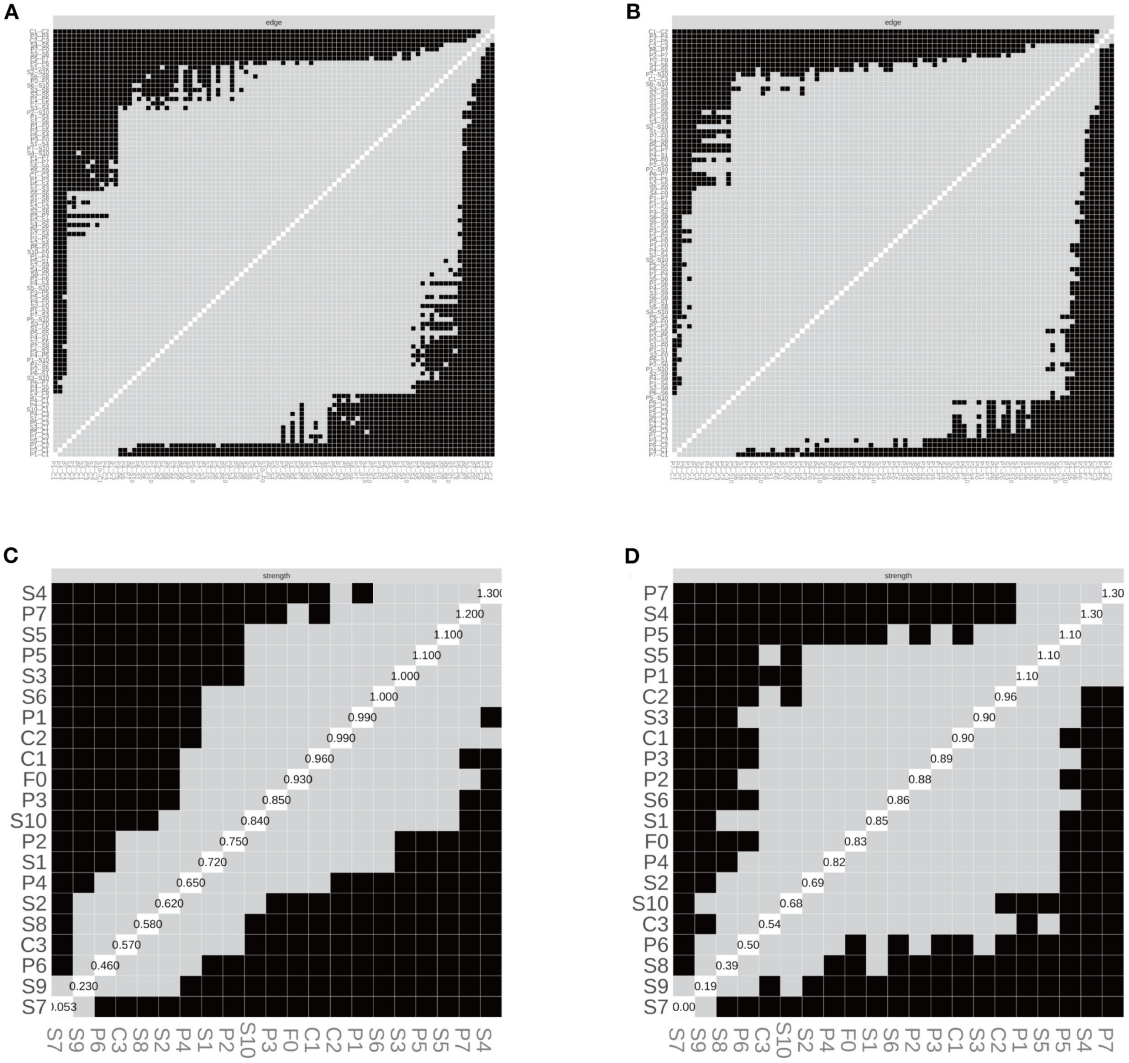
Edge structure and node strength by shift-work subgroup. **(A)** SW—edge-selection matrix; **(B)** NS—edge-selection matrix; **(C)** SW—node-strength heatmap; **(D)** NS—node-strength heatmap. Networks were estimated with EBICglasso (γ = 0.50) on Z-standardized variables, with covariates controlled as specified in the Methods (irregular/on-call schedules excluded). In **(A, B)**, cells denote the presence of non-zero partial-correlation edges (gray = present; black = absent; diagonal = self). In **(C, D)**, shading reflects node strength (lighter = larger), and diagonal labels show each node’s standardized strength value (sum of absolute edge weights). SW = shift-work; NS = non-shift; P1–P7 = PSQI components; S1–S10 = SCL-90 subscales; C1–C3 = CD-RISC factors; F0 = FSS total score. Strength = sum of absolute partial-correlation edge weights.

In the SD group, the correlation stability coefficients (CS) for strength and expected influence (EI) were 0.370 and 0.267, respectively—both exceeding the minimum acceptable threshold of 0.25, indicating adequate stability. Greater centrality stability was observed in the SN group (CS = 0.636), the SW group (Strength CS = 0.596), and the NS group (Strength CS = 0.492). Bootstrap-based confidence intervals for edge weights across all subgroups (**Figures 7**–**10**) indicated overall reliability of network estimation.

### 3.5 Subgroup network comparison

The Network Comparison Test (NCT) suggested significant differences between the SD and SN groups in both structure (M = 0.306, P = 0.003) and global strength (S = 1.012, P = 0.007).

The SN group demonstrated higher overall connectivity (7.053 vs. 6.041), suggesting that individuals with normal sleep exhibited tighter integration and greater propagation potential in the affect–sleep symptom network, whereas sleep disturbance may lead to fragmented connectivity and weakened coupling.

In the shift-work comparison, SW and NS groups differed significantly in network structure (M = 0.295, P = 0.014), but not in global strength (S = 0.122, P = 0.694).

Comparable average edge weights across the two groups suggest that shift status may primarily influence the configuration and layout of connections, rather than the overall magnitude of connectivity, resulting in similar levels of propagation potential.

### 3.6 Sensitivity analyses

**Figure 11:**
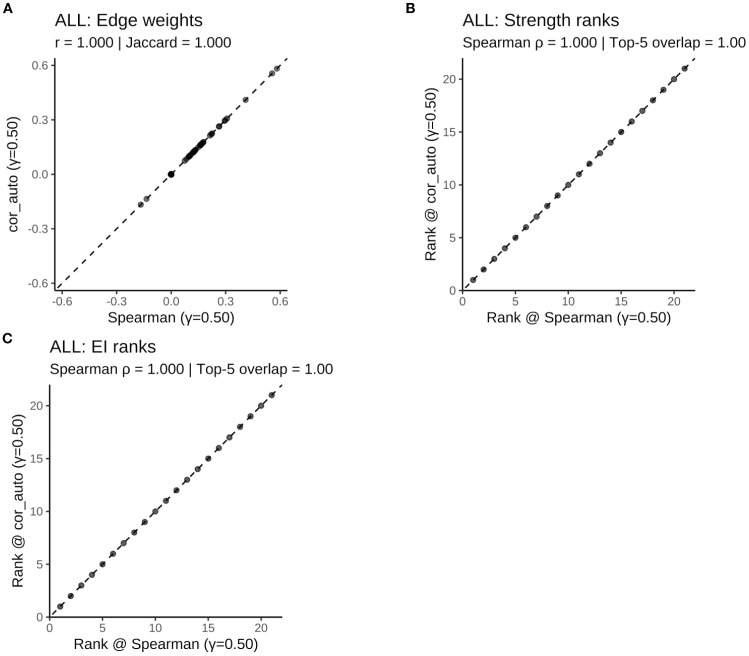
Robustness of the ALL symptom network to correlation estimators (γ = 0.50): Spearman vs. polychoric correlation (cor_auto). **(A)** Scatterplot of edge weights estimated using Spearman versus cor_auto; the dashed line indicates the identity line. **(B)** Comparison of node strength rankings under both correlation methods. **(C)** Comparison of expected influence (EI) rankings under both correlation methods. The two correlation estimators yielded nearly identical networks: edge weights aligned along the 1:1 line, with perfect consistency across all metrics (Pearson r = 1.000, Jaccard = 1.000, sign agreement = 1.000, Spearman ρ = 1.000, Top-5 overlap = 1.00). Results indicate that network structure and centrality rankings are robust to the choice of correlation method.

**Figure 12:**
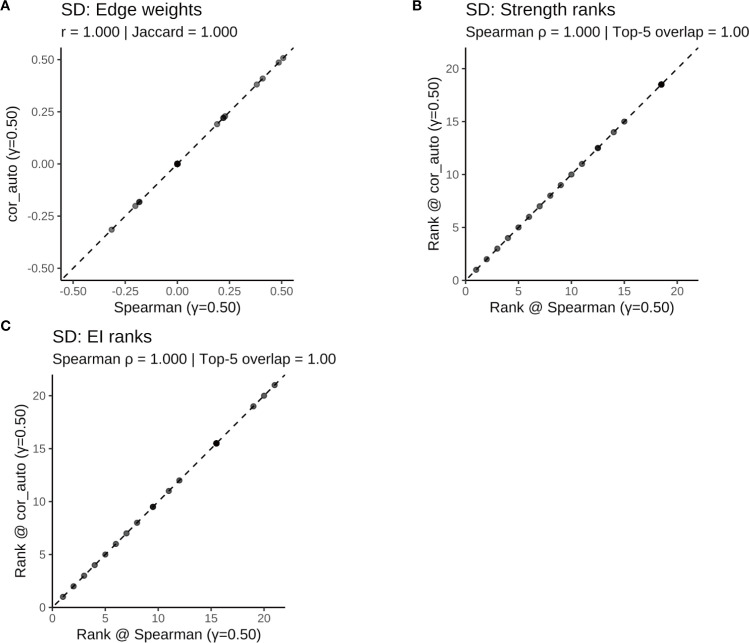
Comparison of network structure and centrality rankings between Spearman and cor_auto methods in the SD subgroup. **(A)** Scatterplot of edge weights estimated using Spearman versus cor_auto; the dashed line indicates the identity line. **(B)** Comparison of node strength rankings under both correlation methods. **(C)** Comparison of expected influence (EI) rankings under both correlation methods. The two correlation estimators yielded nearly identical networks: edge weights aligned along the 1:1 line, with perfect consistency across all metrics (Pearson r = 1.000, Jaccard = 1.000, sign agreement = 1.000, Spearman ρ = 1.000, Top-5 overlap = 1.00). Results indicate that network structure and centrality rankings are robust to the choice of correlation method.

**Figure 13:**
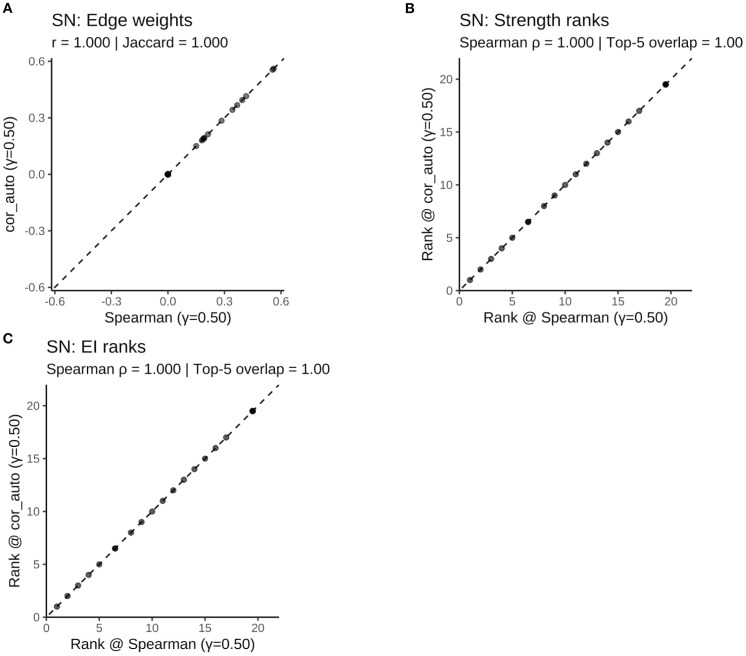
Comparison of network structure and centrality rankings between Spearman and cor_auto methods in the SN subgroup. **(A)** Scatterplot of edge weights estimated using Spearman versus cor_auto; the dashed line indicates the identity line. **(B)** Comparison of node strength rankings under both correlation methods. **(C)** Comparison of expected influence (EI) rankings under both correlation methods. The two correlation estimators yielded nearly identical networks: edge weights aligned along the 1:1 line, with perfect consistency across all metrics (Pearson r = 1.000, Jaccard = 1.000, sign agreement = 1.000, Spearman ρ = 1.000, Top-5 overlap = 1.00). Results indicate that network structure and centrality rankings are robust to the choice of correlation method.

**Figure 14:**
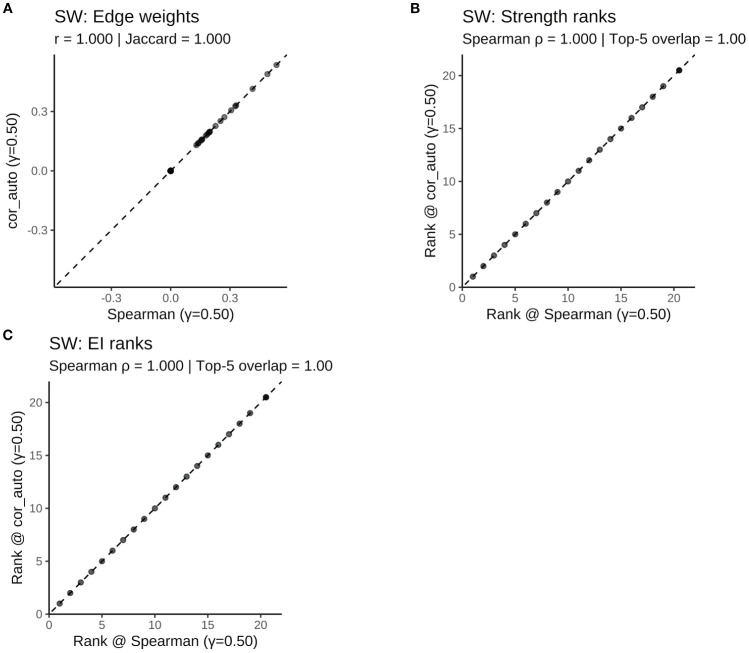
Comparison of network structure and centrality rankings between Spearman and cor_auto methods in the SW subgroup. **(A)** Scatterplot of edge weights estimated using Spearman versus cor_auto; the dashed line indicates the identity line. **(B)** Comparison of node strength rankings under both correlation methods. **(C)** Comparison of expected influence (EI) rankings under both correlation methods. The two correlation estimators yielded nearly identical networks: edge weights aligned along the 1:1 line, with perfect consistency across all metrics (Pearson r = 1.000, Jaccard = 1.000, sign agreement = 1.000, Spearman ρ = 1.000, Top-5 overlap = 1.00). Results indicate that network structure and centrality rankings are robust to the choice of correlation method.

To assess the robustness of the results, two sensitivity analyses were conducted: First, the network was re-estimated using cor_auto (polychoric correlations) instead of Spearman correlations. The resulting network structures were identical across the full sample and all subgroups, with edge weight correlations of r = 1.000, Jaccard similarities = 1.000, and centrality rank correlations (Spearman’s ρ) = 1.000 (**Supplementary Table S5**; **Supplementary Figures S5**–**S9** **S1**; **Figures 11**–**14**). Second, the EBIC hyperparameter γ was varied to 0.25 and 0.75, and compared to the main model (γ = 0.50). Across all γ values, the networks showed high structural consistency, with edge correlations ≥ 0.976, centrality rank correlations (Spearman’s ρ) ≥ 0.966, and substantial overlap in the top 5 central nodes (**Supplementary Table S6**; **Supplementary Figures S2**–**S9**). Although minor fluctuations were observed in network density and global strength, the overall pattern remained stable. Additionally, the top five strongest edges (with bootstrap confidence intervals) were identified for the full and subgroup networks (**Supplementary Table S7**), providing direct estimates of the most robust associations.

Together, these findings confirm that the main results are robust to both analytical methods and tuning parameter choices.

### 3.7 Directed acyclic graph modeling in the sleep-disturbed group

**Figure 15:**
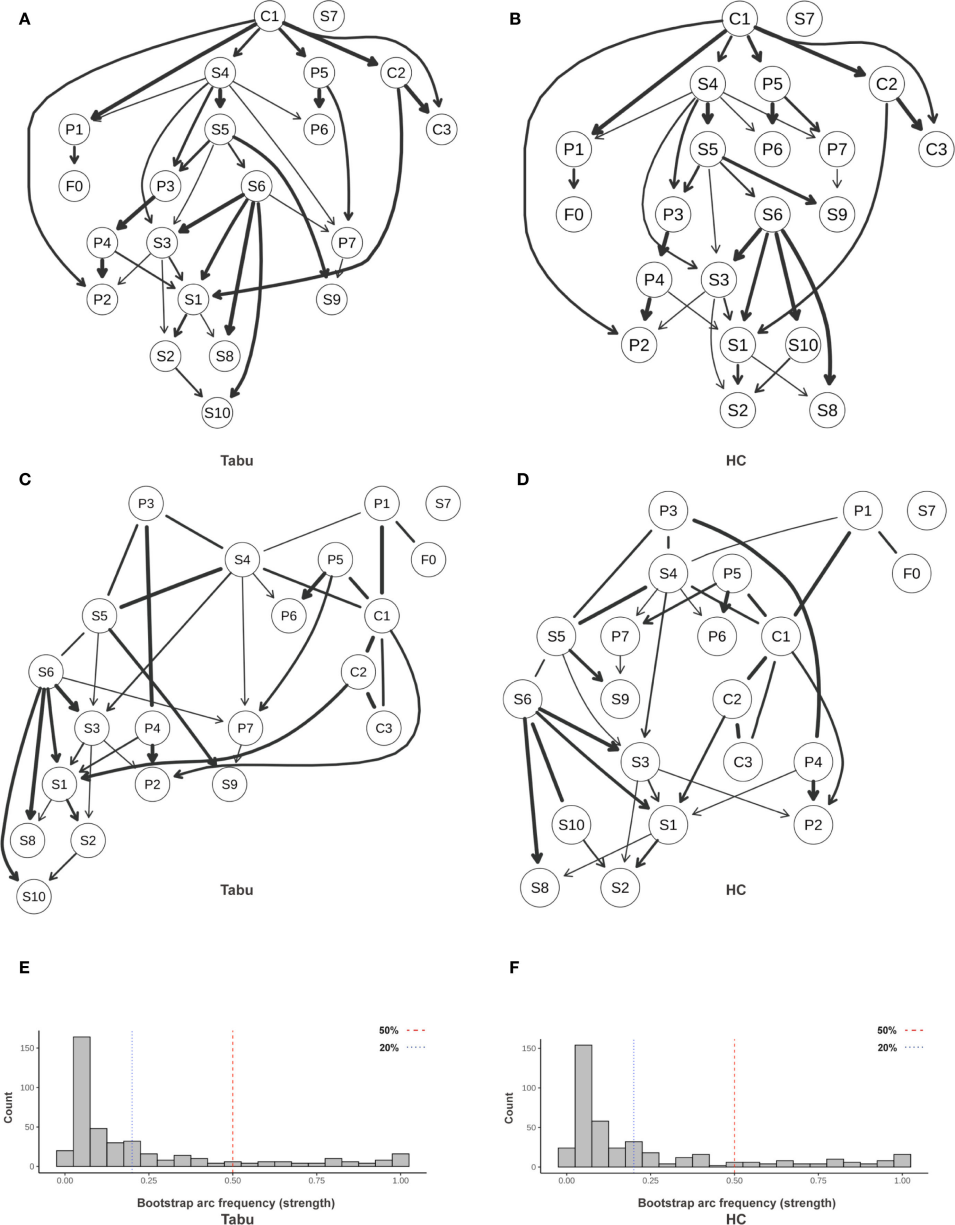
Directed acyclic graph (DAG) illustrating directional relationships among symptoms. **(A)** Averaged DAG (Tabu); **(B)** Averaged DAG (HC); **(C)** CPDAG derived from Tabu; **(D)** CPDAG derived from HC; **(E, F)** Bootstrap arc-frequency (strength) distributions for Tabu and HC. Structures were learned with a BIC score (Gaussian assumption). Nonparametric bootstrap (R = 5,000) was used to compute arc strength (proportion of resamples containing an arc) and direction probability (conditional probability of the shown orientation given arc presence). Edge width = arc strength; darker arrows = higher direction probability; light gray segments = lower support. In **(E, F)**, vertical dashed lines mark reference thresholds (0.20 and 0.50). Variables were Z-standardized prior to learning. Arc strength = bootstrap inclusion proportion; Direction probability = conditional probability of the displayed orientation given presence. SD, sleep-disturbed; Tabu, tabu search; HC, hill-climbing; CPDAG, completed partially directed acyclic graph; P1–P7, PSQI components; S1–S10, SCL-90 subscales; C1–C3, CD-RISC factors; F0, FSS total score.

To explore the directional dependencies among fatigue, sleep, psychological distress, and resilience in individuals with sleep disturbance (SD), we applied parallel structure learning using Tabu and Hill-Climbing (HC) algorithms with Bayesian Information Criterion (BIC) scoring to the residualized SD subgroup (n = 262). Edge strength and directional probabilities were estimated via 5,000 bootstrap replications. A completed partially directed acyclic graph (CPDAG) was generated to account for undirected edges within Markov equivalence classes. The primary threshold for edge inclusion was set at ≥ 0.50 (**Figure 15**; **Supplementary Table S8**), while a sensitivity threshold was set at ≥ 0.20 (**Supplementary Figure S10**; **Supplementary Table S9**).

#### 3.7.1 Primary pathway: subjective sleep → fatigue → depression → hostility

At the primary threshold (edge strength ≥ 0.50), a relatively stable directional chain was identified: subjective sleep quality (P1) → fatigue (F0) → depression (S4) → hostility (S6).

The edge from P1 to F0 showed high algorithmic support, with representative strengths of approximately 0.87 under both Tabu and HC, and directional probabilities > 0.83.

This suggests that poor subjective sleep perception may contribute to increased fatigue.

Likewise, the F0 → S4 connection demonstrated stable support (strength > 0.78), indicating that fatigue may serve as a mediator between nocturnal sleep experience and emotional disturbances.

#### 3.7.2 Upstream protection: cross-domain regulation by hardiness (C1)

The resilience subnetwork revealed a top-down architecture centered on hardiness (C1). Directional edges from C1 to strength (C2) and optimism/control (C3) had near-maximal strength under both algorithms (≈1.00; **Table 1**).

Cross-domain effects included C1 → sleep latency (P2) (strength ≈ 0.80, directionality ≈ 0.92), and C1 → subjective sleep quality (P1) and C1 → depression (S4), both reaching or approaching the primary threshold (strengths ranging from 0.78–0.80).

These findings suggest that hardiness may exert a protective effect by lowering presleep cognitive arousal and negative sleep appraisal, and by directly attenuating upstream activation of depressive symptoms.

In addition, the edge from C2 to somatization (S1) (strength > 0.89) implies that perceived strength may be associated with reduced somatic symptom expression.

#### 3.7.3 Emotional diffusion and downstream somatization

The directed edge from depression (S4) to hostility (S6) (strength > 0.73, directionality > 0.68) suggests a potential affective diffusion pathway from internalizing to externalizing symptoms.

Hostility (S6) further connected to somatization (S1) and other symptoms (S10), with several edge strengths exceeding 0.90, highlighting its potential role as a downstream “amplifier” of emotional dysregulation and somatic expression.

#### 3.7.4 Robustness and uncertainty

Algorithmic consistency was excellent, with near-perfect correlation in edge strengths between Tabu and HC, and high agreement in edge directionality (**Supplementary Tables S8**, **S9**).

Under both primary and sensitivity thresholds, the directional pathways P1 → F0 → S4 → S6 and the upstream C1 module remained stable.

CPDAG results revealed several undirected edges (e.g., P1—S4 or P1—F0), indicating that such relationships lie within a Markov equivalence class and cannot be definitively oriented; these may reflect potential bidirectionality or unmeasured confounding, and should be interpreted as hypothesis-generating.

Regarding structural sparsity, the number of retained directed edges averaged 35–36 under the primary threshold (≥0.50) and 66–70 under the sensitivity threshold (≥0.20) (**Supplementary Table S10**), indicating a moderately sparse yet stable network across algorithms.

## 4 Discussion

This study integrated symptom network analysis with exploratory Directed Acyclic Graph (DAG) modeling to systematically examine the interrelationships among sleep disturbances, fatigue, psychological distress, and resilience in island firefighters. We additionally compared network structures across sleep status (SD vs. SN) and work schedule (SW vs. NS) subgroups. Key findings include: (1) Depression (S4) and daytime dysfunction (P7) showed relatively higher strength and expected influence (EI) in the overall network, suggesting a possible role as symptom hubs; (2) the SD group demonstrated a more activated network structure centered around anxiety and hostility, while the SN group was characterized by internalizing symptoms such as obsessive-compulsion and interpersonal sensitivity; (3) the SW subgroup showed more densely connected sleep–affect pathways, with depressive symptoms and daytime dysfunction occupying the most central positions; and (4) DAG modeling identified a potential directional chain—P1 → F0 → S4 → S6—in the SD group, with resilience (C1) emerging as a possible upstream regulatory node, collectively providing tentative support for a fatigue–sleep–affect cascade pathway.

The prevalence of sleep disturbance among island firefighters in this study reached 46.0%, which is consistent with previous reports (3). In the overall network structure, depression (S4) and daytime dysfunction (P7) ranked among the top in centrality metrics, suggesting that they may function as core transdiagnostic hubs bridging multiple symptom domains. This finding extends prior research in high-stress occupations, where depressive symptoms have been repeatedly identified as convergence points between affective and sleep-related dysfunctions (35, 36). Additional support comes from a network study by Liu et al. on Chinese firefighters, which demonstrated that the “emotional exhaustion” node in burnout was densely connected to various sleep components—potentially acting as a bridge between emotional depletion and emerging sleep problems (37).

Our results further suggest that depressive symptoms may contribute to a cascade of distress and somatization through psychological mechanisms such as hopelessness and self-criticism. Moreover, poor nighttime sleep might exacerbate depression via daytime functional impairments (38, 39). Importantly, daytime dysfunction (P7) may not only be a direct consequence of disrupted sleep, but also serve as a potential mediator linking sleep quality, occupational performance, and negative affect (40). Persistent functional decline might contribute to elevated psychophysiological stress, potentially affecting the hypothalamic–pituitary–adrenal (HPA) axis and circadian regulation, which could in turn perpetuate a maladaptive cycle of “sleep disturbance → impaired functioning → emotional arousal → hypervigilance → further sleep disturbance” (41). Complementary findings from another symptom network study in firefighters revealed plausible directional paths from insomnia components (e.g., subjective sleep quality, sleep latency) to affective symptoms, highlighting the potential role of disturbed sleep perception as an upstream activator of emotional distress (42). Taken together, these findings provide tentative support for our observation that both depression and daytime dysfunction may play influential roles in the network, and underscore their potential relevance as intervention targets.

In the comparison by sleep status, the SD network was centered around anxiety (S5), hostility (S6), and depression (S4), aligning with the hyperarousal model that emphasizes reciprocal amplification among stress, emotion, and sleep dysfunction (43). Individuals with heightened arousal often exhibit increased emotional reactivity and stress sensitivity (44, 45), which may promote frequent activation of negative emotional states (46, 47). Given the highly masculinized culture of firefighting, emotional suppression may be culturally reinforced to conform to masculine norms, thereby potentially leading to somatic manifestations of psychological distress (48–50).

Hostility (S6) emerged as a central node with consistent links to somatization (S1) and other symptoms (S10) across both algorithms, suggesting a possible role as a mediator and amplifier in the downstream diffusion of negative affect, which is also consistent with the emotion suppression hypothesis, wherein hostility is externalized through somatic pathways (37). By contrast, the SN network showed greater centrality for obsessive-compulsion (S2) and interpersonal sensitivity (S3), which may reflect a greater reliance on internal control and social regulation in better-sleeping individuals—an indirect indication that emotional–sleep interference might be more prominent in the SD group.

In the comparison by work schedule, the SW network displayed denser and more direct sleep–affect connectivity, with depression (S4) and daytime dysfunction (P7) occupying central positions, whereas the NS network placed relatively greater emphasis on sleep dimensions such as subjective sleep quality (P1) and sleep disturbance (P5). This pattern aligns with prior evidence indicating that shift work may be associated with circadian disruption and altered melatonin secretion, potentially increasing vulnerability to neuroendocrine–immune dysregulation (13, 51, 52). Shift workers in healthcare and emergency services consistently report higher rates of sleep problems, cognitive hyperarousal, and affective disturbances (53). These findings suggest the potential value of implementing circadian-informed strategies—such as optimized shift scheduling and light-based interventions—to promote sleep health in shift-working populations.

The DAG modeling in the SD subgroup provided preliminary directional insight, identifying a possible progressive chain of P1 → F0 → S4 → S6, as well as upstream influences from resilience (C1) targeting P1, P2, and S4, all offering initial support for a hypothesized fatigue–sleep–affect progression model. From a neurobiological perspective, chronic fatigue has been linked to the accumulation of neurotoxic metabolites (39) and elevated oxidative stress, which may disrupt glutamate–glutamine cycling and limbic–prefrontal regulatory circuits (54–58). Such dysregulation can activate the HPA axis and pro-inflammatory cytokines, leading to disturbances in cortisol circadian rhythms (59, 60), which in turn may contribute to fragmented sleep architecture, emotional hyperarousal, and somatic symptoms (61, 62).

Resilience (C1) occupied an upstream position in the DAG, pointing to strength (C2), optimism/control (C3), and multiple sleep/emotion nodes. This configuration suggests that C1 may function as an upstream regulatory factor in the fatigue–sleep–emotion cascade, potentially modulating presleep cognitive arousal and negative sleep appraisal to enhance emotional stability (17). When this psychological resource system is impaired, the risk of maladaptive emotion–sleep interactions may escalate, highlighting the potential utility of early resilience-based interventions aimed at strengthening subjective control and reducing sleep-related cognitive arousal.

Based on the integrated findings from the network and DAG analyses, we propose several preliminary intervention targets and pathways: (1) For high-impact nodes such as depression (S4) and daytime dysfunction (P7), behavioral activation and emotion regulation training may help mitigate downstream symptom spread. (2) For prominent nodes in the sleep-disturbed group—namely anxiety (S5) and hostility (S6)—relaxation training and expressive emotion interventions might interrupt the hyperarousal–sleep–affect cycle. (3) To address the intensified sleep–depression link in shift workers, circadian-based interventions such as optimized scheduling, light therapy, and melatonin regulation could be prioritized at both organizational and individual levels. (4) Given C1’s upstream position in the resilience architecture, stress management and self-efficacy training may enhance its buffering capacity, providing a potential theoretical basis for tailored intervention planning.

## 5 Limitations

This study has several limitations that warrant consideration. First, the cross-sectional design restricts causal inference and limits the ability to determine temporal precedence. While DAG models were employed to explore potential directional dependencies, they reflect conditional associations rather than causality, and key pathways require validation through longitudinal or interventional studies. Second, all variables were measured using self-report instruments, which may be subject to biases related to social desirability or cognitive style. Future studies could incorporate clinician-rated scales and objective indicators (e.g., actigraphy-based circadian or sleep assessments). Third, the sample was predominantly male, with a low proportion of female participants, limiting the generalizability of findings across gender. Future work should aim to increase female representation and perform gender-stratified analyses. Fourth, data collection was concentrated in July 2023—a period characterized by elevated heat and operational demand in island regions—introducing potential seasonal and workload-related biases. Fifth, for model parsimony and stability, this study focused on factor/construct-level nodes rather than individual items. This may obscure within-domain heterogeneity, and future research is needed to validate item-level symptom networks.

## 6 Conclusions

This study employed network psychometrics and exploratory DAG modeling to delineate the interrelations and potential directional patterns among sleep disturbances, fatigue, psychological distress, and resilience in island firefighters. Depression (S4) and daytime dysfunction (P7) appeared to function as central symptoms, while fatigue (F0) might act as a bridge between subjective sleep perception and affective symptoms. Although the overall network strength remained relatively stable across sleep and shiftwork subgroups, the structural layout of symptom connectivity varied modestly. DAG modeling suggested a tentative directional chain from subjective sleep quality to emotional disturbances (P1 → F0 → S4 → S6), with resilience (C1) occupying a potential upstream position and showing directed links toward P1, P2, and S4—pointing to a possible buffering pathway.

These findings may offer a novel network-informed perspective to understand the dynamics of sleep–psychological comorbidity in high-risk occupational groups and may help inform theoretical frameworks for targeted interventions. Future research should incorporate longitudinal designs and objective metrics to further explore the causal nature of key pathways and support the development of multi-level strategies addressing core symptoms (e.g., depression, daytime dysfunction) and upstream resilience factors.

## Data Availability

The raw data supporting the conclusions of this article will be made available by the authors, without undue reservation.

## Supplementary material

Supplementary Table 1 Baseline Demographic and Lifestyle Characteristics by PSQI Group (n = 570). Values are presented as median (Q1, Q3) for continuous variables and n (%) for categorical variables. P-values were calculated using Mann–Whitney U test (a), Pearson’s χ² test (b), or Fisher’s exact test (c) as appropriate. PSQI > 7 = Sleep-disturbed group; PSQI ≤ 7 = Sleep-normal group. Bold p-values indicate statistical significance at α = 0.05.

Supplementary Table 2 Baseline Demographic and Lifestyle Characteristics by Work Schedule (n = 432). Values are presented as median (Q1, Q3) for continuous variables and n (%) for categorical variables. P-values were calculated using Mann–Whitney U test (a), Pearson’s χ² test (b), or Fisher’s exact test (c) as appropriate. SW, Shift-work group; NS, Non-shift group. Bold p-values indicate statistical significance at α = 0.05.

Supplementary Table 3 Symptom Differences Between Sleep-Disturbed and Sleep-Normal Firefighters (n = 570). Values are expressed as median (Q1, Q3). Group comparisons were performed using Mann–Whitney *U* tests due to non-normal distribution of most variables. SD group = Sleep-disturbed group (PSQI > 7); SN group = Sleep-normal group (PSQI ≤ 7). P = PSQI components (P1–P7); S = SCL-90 subscales (S1–S10); C = CD-RISC factors (C1–C3); F = FSS total score. All *p*-values are two-tailed; values in bold indicate statistical significance at α = 0.05.

Supplementary Table 4 Symptom Differences Between Shift-Work and Non-Shift Firefighters (n = 432). Values are expressed as median (Q1, Q3). Group comparisons were performed using Mann–Whitney *U* tests due to non-normal distribution of most variables. SD = Sleep-disturbed group (PSQI > 7); SN = Sleep-normal group (PSQI ≤ 7); P = PSQI components (P1–P7); S = SCL-90 subscales (S1–S10); C = CD-RISC factors (C1–C3); F = FSS total score. All *p*-values are two-tailed; values in bold indicate statistical significance at α = 0.05.

Supplementary Table 5 Robustness of Symptom Networks to Correlation Estimators (Spearman vs cor_auto; γ = 0.50). ALL = full sample; SN = sleep-normal; SD = sleep-disturbed; NS = non-shift; SW = shift-work; EI = Expected Influence. Networks were estimated with EBICglasso at γ = 0.50. “Spearman” uses rank correlations; “cor_auto” uses polychoric/tetrachoric correlations for ordinal items. Density = proportion of non-zero edges; Global strength = sum of absolute edge weights; Top-k overlap = proportion of top-k nodes (by Strength) preserved across estimators.

Supplementary Table 6 Structural and Centrality Stability of Symptom Networks Under Varying EBIC Hyperparameters (γ = 0.25, 0.50, 0.75). This table summarizes the structural and centrality stability metrics of the estimated symptom networks under varying EBIC hyperparameter values (γ = 0.25, 0.50, 0.75). The reference network is estimated using γ = 0.50, while γ = 0.25 and γ = 0.75 serve as sensitivity comparisons. SD, sleep-disturbed; SN, sleep-normal; SW, shift-work; NS, non-shift; EI, Expected Influence; Jaccard, Jaccard similarity index of edge presence; Edge r, Pearson correlation of edge weights; Sign Agreement, proportion of consistent edge signs; ρ (Strength) and ρ (EI), Spearman correlations of node-level strength and expected influence; Density, proportion of nonzero edges; GStrength, Global Strength (sum of absolute edge weights); Top-k, proportion of top-k ranked nodes (k = 3, 5, 10) preserved across networks.

Supplementary Table 7 Top 5 Strongest Edges and 95% Confidence Intervals in Each Subgroup Network. This table lists the top 5 edges with the highest absolute weights in each estimated network (overall and subgroups). The edge weight reflects the strength of association between nodes. 95% nonparametric bootstrap confidence intervals were computed for each edge. SD, sleep-disturbed group; SN, sleep-normal group; SW, shift-work group; NS, non-shift group.

Supplementary Table 8 Comparison of Edge Strength and Directional Consistency Between Tabu and HC Algorithms (γ = 0.50). This table compares the edge strength and directional probabilities of arcs identified by the Tabu and Hill-Climbing (HC) algorithms in the estimated Bayesian DAG (Directed Acyclic Graph) models (bootstrap repetitions R = 5000). “Arc” denotes the estimated directed edge between two nodes. “Strength” represents the bootstrap frequency of arc presence, and “Direction” denotes the estimated directional probability of each arc. “Agreement” indicates whether both algorithms identified the same arc with consistent direction. Only arcs with bootstrap presence frequency ≥ 0.50 are included.

Supplementary Table 9 Comparison of Edge Strength and Directional Consistency Between Tabu and HC Algorithms (γ = 0.20). This table compares the edge strength and directional probabilities of arcs identified by the Tabu and Hill-Climbing (HC) algorithms in the estimated Bayesian DAG (Directed Acyclic Graph) models (bootstrap repetitions R = 5000). “Arc” denotes the estimated directed edge between two nodes. “Strength” represents the bootstrap frequency of arc presence, and “Direction” denotes the estimated directional probability of each arc. “Agreement” indicates whether both algorithms identified the same arc with consistent direction. Only arcs with bootstrap presence frequency ≥ 0.50 are included.

Supplementary Table 10 Number of Arcs Retained under Different Bootstrap Thresholds and Algorithms in the Sleep Disturbance Subgroup (n=262). This table summarizes the number of node pairs (edges) and directed arcs retained in the averaged DAGs, generated using 5,000 bootstrap samples. Two structure learning algorithms (Tabu and Hill-Climbing) were applied, and two arc frequency thresholds were tested (≥0.50 and ≥0.20). “Edges Retained” refers to the number of node pairs with arc frequency exceeding the specified threshold; “Directed Arcs” refers to the number of directed edges retained in the final averaged DAG under each condition.

## Glossary

PSQI: Pittsburgh Sleep Quality Index
FSS: Fatigue Severity Scale
SCL-90: Symptom Checklist-90
CD-RISC: Connor–Davidson Resilience Scale
SNA: Symptom Network Analysis
DAG: Directed Acyclic Graph
CPDAG: Completed Partially Directed Acyclic Graph
GGM: Gaussian Graphical Model
MGM: Mixed Graphical Model
EBICglasso: Extended BIC Graphical LASSO
BIC: Bayesian Information Criterion
HC: Hill-Climbing
Tabu: Tabu Search
NCT: Network Comparison Test
EI: Expected Influence
Strength: Strength centrality
R²: Predictability (explained variance)
CS: Correlation Stability
CI: Confidence Interval
α: Cronbach’s alpha
SD/SN: Sleep-Disturbed/Sleep-Normal
SW/NS: Shift-Work/Non-Shift
HPA: Hypothalamic–Pituitary–Adrenal axis
P1: PSQI – Subjective Sleep Quality
P2: PSQI – Sleep Latency
P3: PSQI – Sleep Duration
P4: PSQI – Habitual Sleep Efficiency
P5: PSQI – Sleep Disturbances
P6: PSQI – Use of Sleeping Medication
P7: PSQI – Daytime Dysfunction
S1: SCL-90 – Somatization
S2: SCL-90 – Obsessive–Compulsive
S3: SCL-90 – Interpersonal Sensitivity
S4: SCL-90 – Depression
S5: SCL-90 – Anxiety
S6: SCL-90 – Hostility
S7: SCL-90 – Phobic Anxiety
S8: SCL-90 – Paranoid Ideation
S9: SCL-90 – Psychoticism
S10: SCL-90 – Additional Items
C1: CD-RISC – Tenacity/Hardiness
C2: CD-RISC – Personal Strength
C3: CD-RISC – Optimism & Control
F0: FSS – Total Score
