# Supplementary material for: Depression and daytime dysfunction centralize the fatigue–sleep cascade in island firefighters: a symptom network and Bayesian DAG study
Source: Front Psychiatry. 2025 Oct 29;16:1663957. doi: 10.3389/fpsyt.2025.1663957 (PMC12605024; doi:10.3389/fpsyt.2025.1663957)
Supplement: Supplementary Table 1 — Baseline Demographic and Lifestyle Characteristics by PSQI Group (n = 570). Values are presented as median (Q1, Q3) for continuous variables and n (%) for categorical variables. P-values were calculated using Mann–Whitney U test (a), Pearson’s χ² test (b), or Fisher’s exact test (c) as appropriate. PSQI > 7 = Sleep-disturbed group; PSQI ≤ 7 = Sleep-normal group. Bold p-values indicate statistical significance at α = 0.05. [file DataSheet1.zip › Data Sheet 1/Supplementary Figure Captions.DOCX]

**Supplementary Figure S1.** **Comparison of network structure and centrality rankings between Spearman and cor_auto methods in the NS subgroup**.

**Panels:** (A) Scatterplot of edge weights estimated using Spearman versus cor_auto; the dashed line indicates the identity line. (B) Comparison of node strength rankings under both correlation methods. (C) Comparison of expected influence (EI) rankings under both correlation methods.

**Note.** The two correlation estimators yielded nearly identical networks: edge weights aligned along the 1:1 line, with perfect consistency across all metrics (Pearson r = 1.000, Jaccard = 1.000, sign agreement = 1.000, Spearman ρ = 1.000, Top-5 overlap = 1.00). Results indicate that network structure and centrality rankings are robust to the choice of correlation method.

**Supplementary Figure S2. EBIC γ-sensitivity of network estimates in the full sample (N=570).**

**Panels:** (A) Edge weights: γ = 0.50 vs 0.25; (B) Edge weights: γ = 0.50 vs 0.75; (C) Strength ranks: γ = 0.50 vs 0.25; (D) Strength ranks: γ = 0.50 vs 0.75; (E) Expected influence (EI) ranks: γ = 0.50 vs 0.25; (F) EI ranks: γ = 0.50 vs 0.75.

**Note.** Networks were estimated using EBICglasso on Z-standardized variables (covariates controlled as specified in the Methods). Scatterplots compare estimates at the reference γ = 0.50 with alternative γ values; the dashed line is the identity line. For edge weights (A–B), r is the Pearson correlation and Jaccard is the overlap of non-zero edges between networks. For node centrality (C–F), Spearman ρ quantifies rank correlation and Top-5 overlap is the proportion of nodes shared in the five highest-ranked positions. EBICglasso = extended Bayesian information criterion graphical lasso; γ = EBIC hyperparameter; EI = expected influence.

**Supplementary Figure S3. Network-level metrics by EBIC γ in the full sample (N = 570).**

**Panels:** (A) Network density across γ; (B) Global strength across γ.

**Note.** Networks were estimated using EBICglasso at γ = 0.25, 0.50, and 0.75 on Z-standardized variables, with covariates controlled as described in the Methods. Bars display metric values for each γ. Network density = proportion of non-zero partial-correlation edges among all possible undirected edges; Global strength = sum of absolute partial-correlation edge weights in the network. EBICglasso = extended Bayesian information criterion graphical lasso; γ = EBIC hyperparameter.

**Supplementary Figure S4. EBIC γ-sensitivity of network estimates in the sleep-disturbed subgroup (SD; n = 262).**

**Panels:** (A) Edge weights: γ = 0.50 vs 0.25; (B) Edge weights: γ = 0.50 vs 0.75; (C) Strength ranks: γ = 0.50 vs 0.25; (D) Strength ranks: γ = 0.50 vs 0.75; (E) Expected influence (EI) ranks: γ = 0.50 vs 0.25; (F) EI ranks: γ = 0.50 vs 0.75.

**Note.** Networks were estimated using EBICglasso on Z-standardized variables with covariates controlled (see Methods). Scatterplots compare estimates at the reference γ = 0.50 with alternative γ values; the dashed line is the identity line. For edge weights (A–B), r = Pearson correlation and Jaccard = overlap of non-zero edges between networks. For node centrality (C–F), Spearman ρ = rank correlation and Top-5 overlap = proportion of nodes shared in the five highest-ranked positions. SD = sleep-disturbed; EBICglasso = extended Bayesian information criterion graphical lasso; γ = EBIC hyperparameter; EI = expected influence.

**Supplementary Figure S5. EBIC γ-sensitivity of network estimates in the sleep-normal subgroup (SN; n = 308).**

**Panels:** (A) Edge weights: γ = 0.50 vs 0.25; (B) Edge weights: γ = 0.50 vs 0.75; (C) Strength ranks: γ = 0.50 vs 0.25; (D) Strength ranks: γ = 0.50 vs 0.75; (E) Expected influence (EI) ranks: γ = 0.50 vs 0.25; (F) EI ranks: γ = 0.50 vs 0.75.
**Note.** Networks were estimated using EBICglasso on Z-standardized variables with covariates controlled (see Methods). Scatterplots compare estimates at the reference γ = 0.50 with alternative γ values; the dashed line is the identity line. For edge weights (A–B), r = Pearson correlation and Jaccard = overlap of non-zero edges between networks. For node centrality (C–F), Spearman ρ = rank correlation and Top-5 overlap = proportion of nodes shared in the five highest-ranked positions. SN = sleep-normal; EBICglasso = extended Bayesian information criterion graphical lasso; γ = EBIC hyperparameter; EI = expected influence.

**Supplementary Figure S6. Network-level metrics by EBIC γ in sleep-status subgroups.**

**Panels:** (A) SD—network density by γ; (B) SD—global strength by γ; (C) SN—network density by γ; (D) SN—global strength by γ.

**Note.** Networks were estimated using EBICglasso at γ = 0.25, 0.50, and 0.75 on Z-standardized variables, with covariates controlled as described in the Methods. Bars show metric values at each γ. Network density = proportion of non-zero partial-correlation edges among all possible undirected edges; Global strength = sum of absolute partial-correlation edge weights across the network. SD = sleep-disturbed; SN = sleep-normal; EBICglasso = extended Bayesian information criterion graphical lasso; γ = EBIC hyperparameter.

**Supplementary Figure S7. EBIC γ-sensitivity of network estimates in the shift-work subgroup (SW; n = 255).**

**Panels:** (A) Edge weights: γ = 0.50 vs 0.25; (B) Edge weights: γ = 0.50 vs 0.75; (C) Strength ranks: γ = 0.50 vs 0.25; (D) Strength ranks: γ = 0.50 vs 0.75; (E) Expected influence (EI) ranks: γ = 0.50 vs 0.25; (F) EI ranks: γ = 0.50 vs 0.75.

**Note.** Networks were estimated using EBICglasso on Z-standardized variables with covariates controlled (see Methods). Scatterplots compare estimates at the reference γ = 0.50 with alternative γ values; the dashed line is the identity line. For edge weights (A–B), r = Pearson correlation and Jaccard = overlap of non-zero edges between networks. For node centrality (C–F), Spearman ρ = rank correlation and Top-5 overlap = proportion of nodes shared in the five highest-ranked positions. SW = shift-work; EBICglasso = extended Bayesian information criterion graphical lasso; γ = EBIC hyperparameter; EI = expected influence.

**Supplementary Figure S8. EBIC γ-sensitivity of network estimates in the non-shift subgroup (NS; n = 177).**

**Panels:** (A) Edge weights: γ = 0.50 vs 0.25; (B) Edge weights: γ = 0.50 vs 0.75; (C) Strength ranks: γ = 0.50 vs 0.25; (D) Strength ranks: γ = 0.50 vs 0.75; (E) Expected influence (EI) ranks: γ = 0.50 vs 0.25; (F) EI ranks: γ = 0.50 vs 0.75.

**Note.** Networks were estimated using EBICglasso on Z-standardized variables, with covariates controlled as described in the Methods (shift-status analyses exclude irregular/on-call schedules). Scatterplots compare estimates at the reference γ = 0.50 with alternative γ values; the dashed line is the identity line. For edge weights (A–B), r = Pearson correlation and Jaccard = overlap of non-zero edges between networks. For node centrality (C–F), Spearman ρ = rank correlation and Top-5 overlap = proportion of nodes shared in the five highest-ranked positions. NS = non-shift; EBICglasso = extended Bayesian information criterion graphical lasso; γ = EBIC hyperparameter; EI = expected influence.

**Supplementary Figure S9. Network-level metrics by EBIC γ in shift-work subgroups.**

**Panels:** (A) SW—network density by γ; (B) SW—global strength by γ; (C) NS—network density by γ; (D) NS—global strength by γ.

**Note.** Networks were estimated using EBICglasso at γ = 0.25, 0.50, and 0.75 on Z-standardized variables, with covariates controlled as described in the Methods (irregular/on-call schedules excluded). Bars show the metric value at each γ. Network density = proportion of non-zero partial-correlation edges among all possible undirected edges; Global strength = sum of absolute partial-correlation edge weights across the network. SW = shift-work; NS = non-shift; EBICglasso = extended Bayesian information criterion graphical lasso; γ = EBIC hyperparameter.

**Supplementary Figure S10. CPDAGs of the SD subgroup under a relaxed bootstrap threshold (strength ≥ 0.20).**

**Panels:** (A) Tabu—averaged CPDAG; (B) Hill-Climbing (HC)—averaged CPDAG.

**Note.** Graphs were learned in the sleep-disturbed (SD) group using Bayesian network structure learning with BIC scoring and 5,000 bootstraps. Shown are CPDAGs, where directed arrows indicate compelled directions shared within the Markov equivalence class and undirected connections mark edges whose orientation cannot be identified. Edge thickness reflects the bootstrap arc strength (frequency). Layout was kept consistent across panels to aid comparison. These DAG results are exploratory and hypothesis-generating. SD = sleep-disturbed; Tabu/HC = search algorithms; P1–P7 = PSQI components; S1–S10 = SCL-90 subscales; C1–C3 = CD-RISC factors; F0 = FSS total score.
