# Supplementary figures and images for: Depression and daytime dysfunction centralize the fatigue–sleep cascade in island firefighters: a symptom network and Bayesian DAG study
Source: Front Psychiatry. 2025 Oct 29;16:1663957. doi: 10.3389/fpsyt.2025.1663957 (PMC12605024; doi:10.3389/fpsyt.2025.1663957)

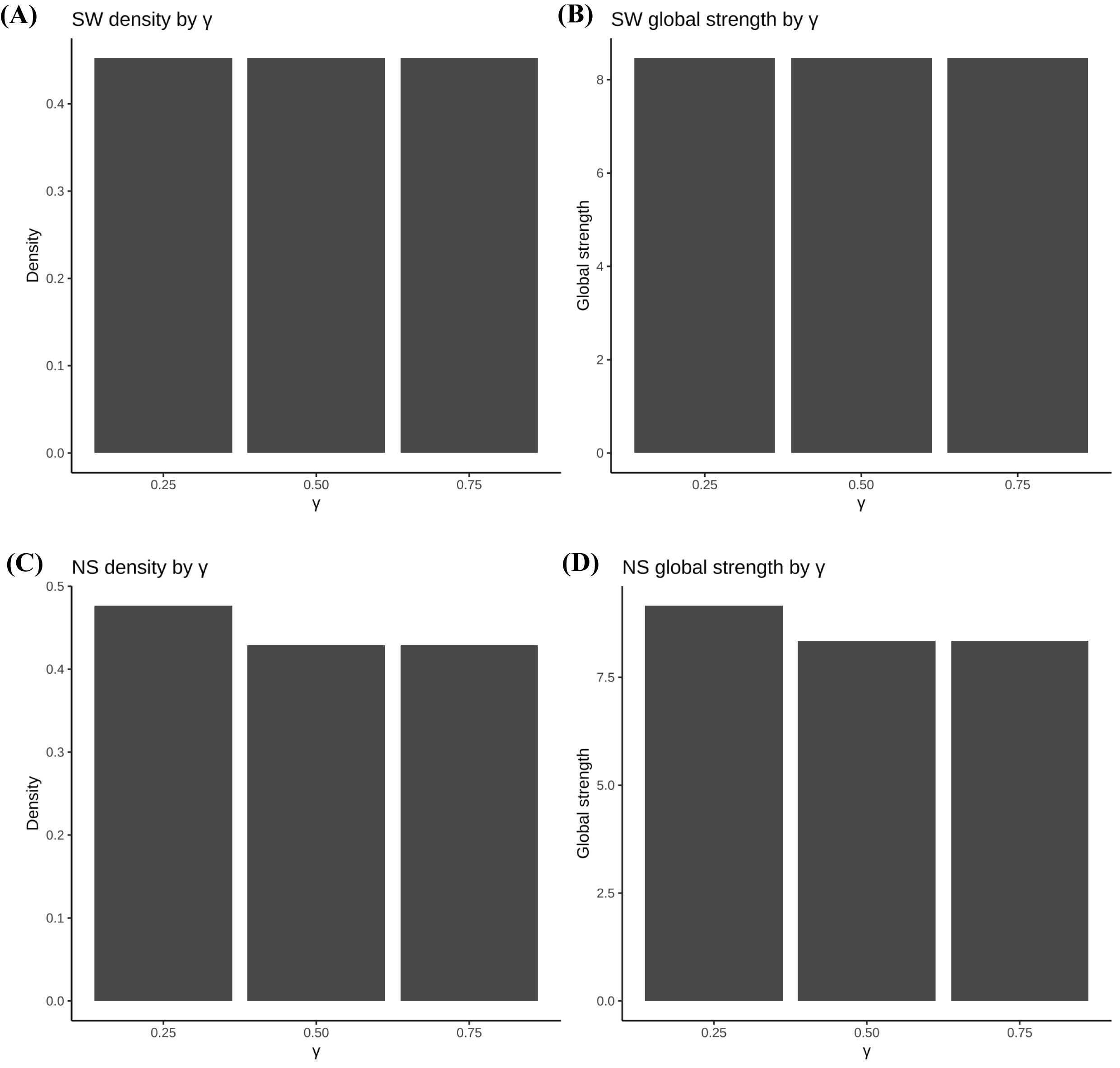

Supplement: Supplementary Table 1 — Baseline Demographic and Lifestyle Characteristics by PSQI Group (n = 570). Values are presented as median (Q1, Q3) for continuous variables and n (%) for categorical variables. P-values were calculated using Mann–Whitney U test (a), Pearson’s χ² test (b), or Fisher’s exact test (c) as appropriate. PSQI > 7 = Sleep-disturbed group; PSQI ≤ 7 = Sleep-normal group. Bold p-values indicate statistical significance at α = 0.05. [file DataSheet1.zip › Data Sheet 1/Figure S9TIF.TIF]

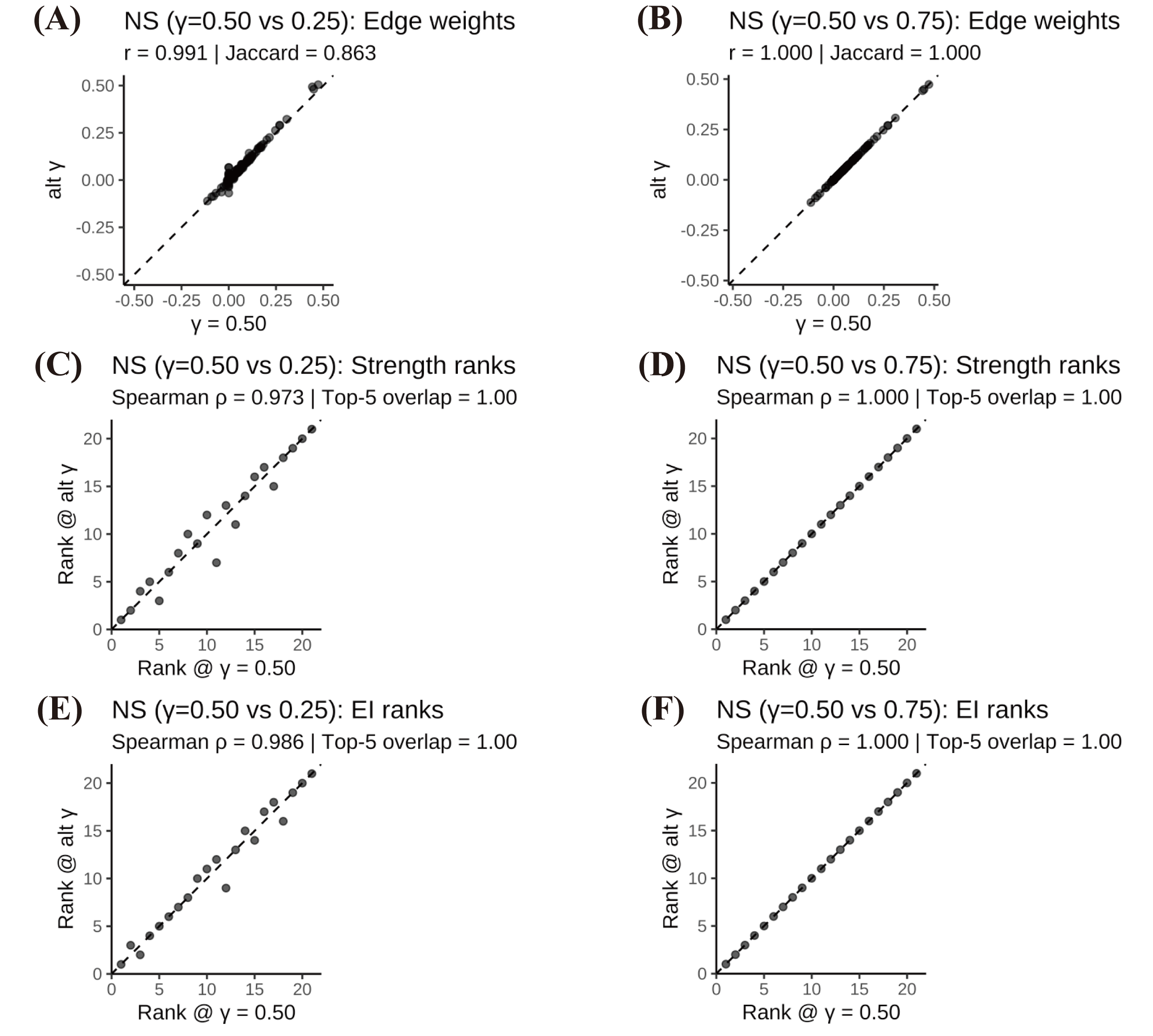

Supplement: Supplementary Table 1 — Baseline Demographic and Lifestyle Characteristics by PSQI Group (n = 570). Values are presented as median (Q1, Q3) for continuous variables and n (%) for categorical variables. P-values were calculated using Mann–Whitney U test (a), Pearson’s χ² test (b), or Fisher’s exact test (c) as appropriate. PSQI > 7 = Sleep-disturbed group; PSQI ≤ 7 = Sleep-normal group. Bold p-values indicate statistical significance at α = 0.05. [file DataSheet1.zip › Data Sheet 1/Figure S8.TIF]

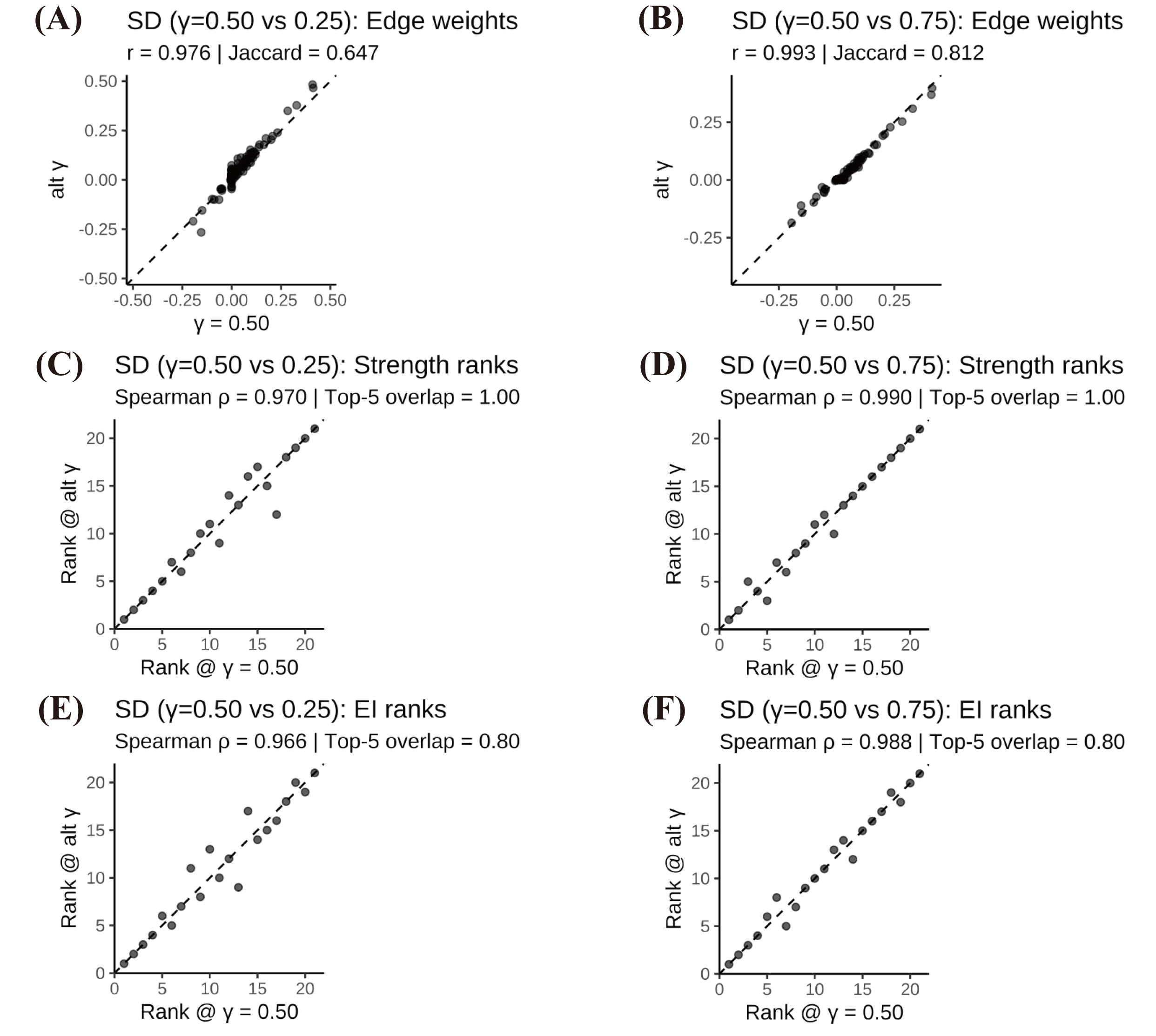

Supplement: Supplementary Table 1 — Baseline Demographic and Lifestyle Characteristics by PSQI Group (n = 570). Values are presented as median (Q1, Q3) for continuous variables and n (%) for categorical variables. P-values were calculated using Mann–Whitney U test (a), Pearson’s χ² test (b), or Fisher’s exact test (c) as appropriate. PSQI > 7 = Sleep-disturbed group; PSQI ≤ 7 = Sleep-normal group. Bold p-values indicate statistical significance at α = 0.05. [file DataSheet1.zip › Data Sheet 1/Figure S4.TIF]

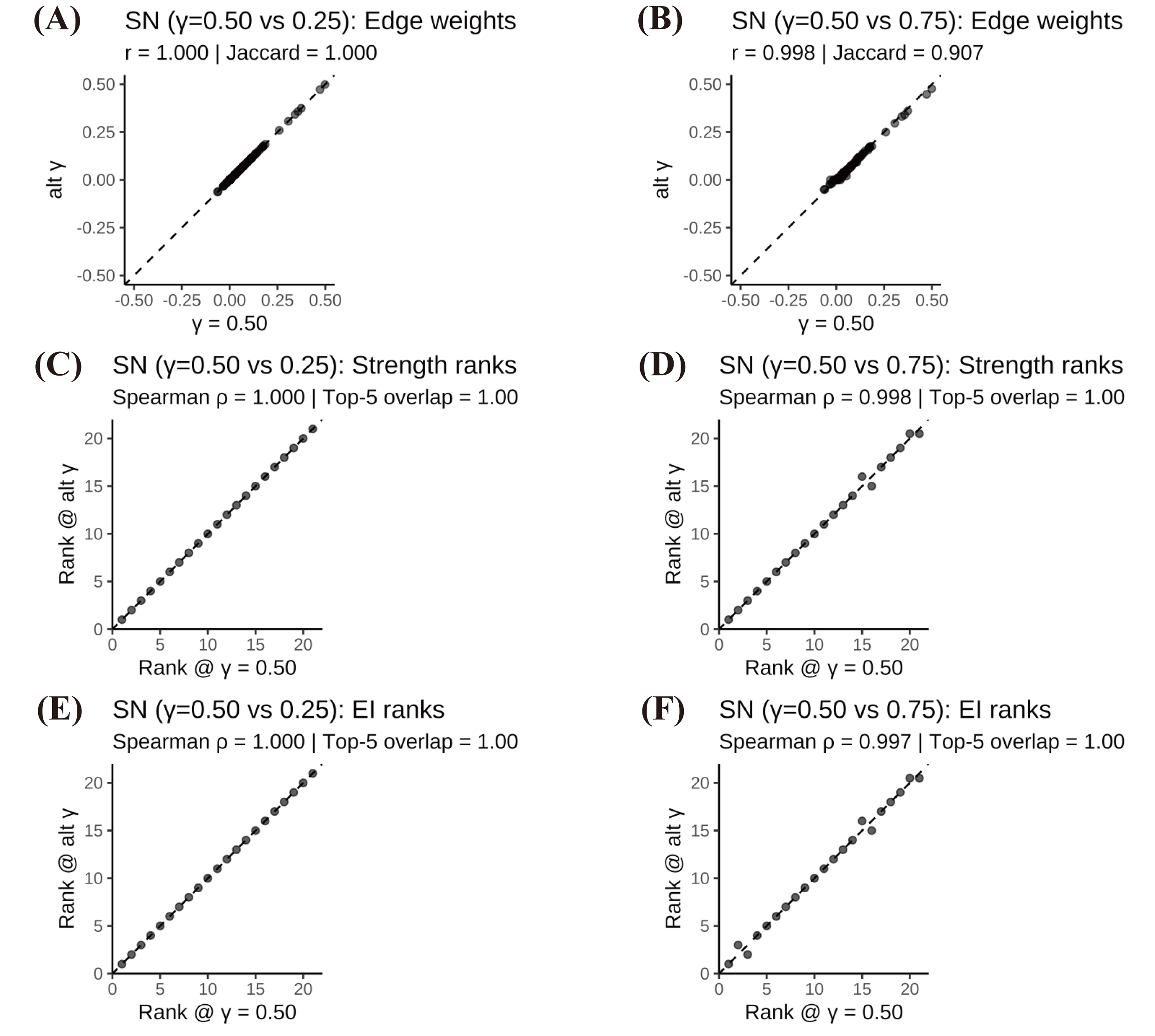

Supplement: Supplementary Table 1 — Baseline Demographic and Lifestyle Characteristics by PSQI Group (n = 570). Values are presented as median (Q1, Q3) for continuous variables and n (%) for categorical variables. P-values were calculated using Mann–Whitney U test (a), Pearson’s χ² test (b), or Fisher’s exact test (c) as appropriate. PSQI > 7 = Sleep-disturbed group; PSQI ≤ 7 = Sleep-normal group. Bold p-values indicate statistical significance at α = 0.05. [file DataSheet1.zip › Data Sheet 1/Figure S5.TIF]

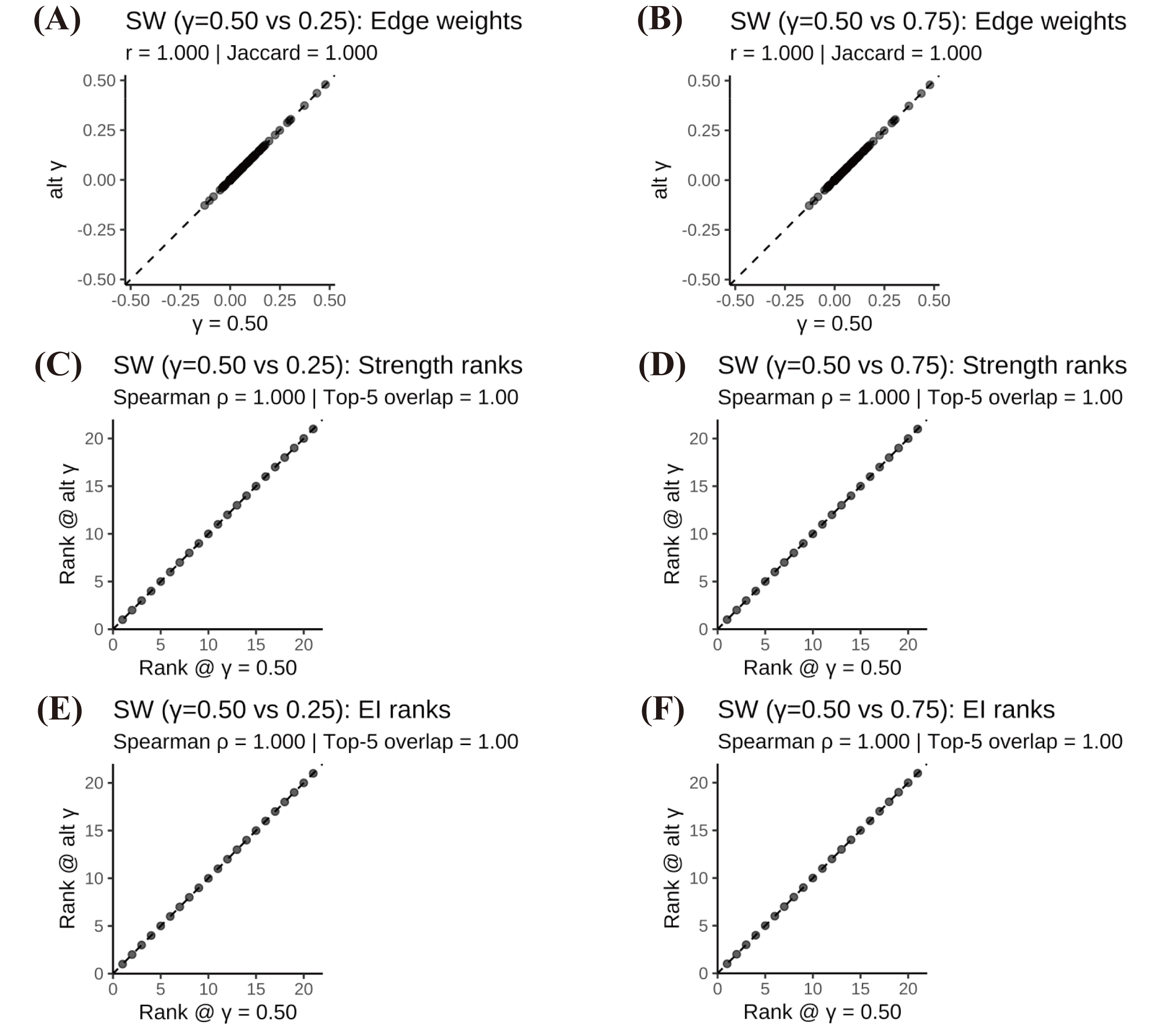

Supplement: Supplementary Table 1 — Baseline Demographic and Lifestyle Characteristics by PSQI Group (n = 570). Values are presented as median (Q1, Q3) for continuous variables and n (%) for categorical variables. P-values were calculated using Mann–Whitney U test (a), Pearson’s χ² test (b), or Fisher’s exact test (c) as appropriate. PSQI > 7 = Sleep-disturbed group; PSQI ≤ 7 = Sleep-normal group. Bold p-values indicate statistical significance at α = 0.05. [file DataSheet1.zip › Data Sheet 1/Figure S7.TIF]

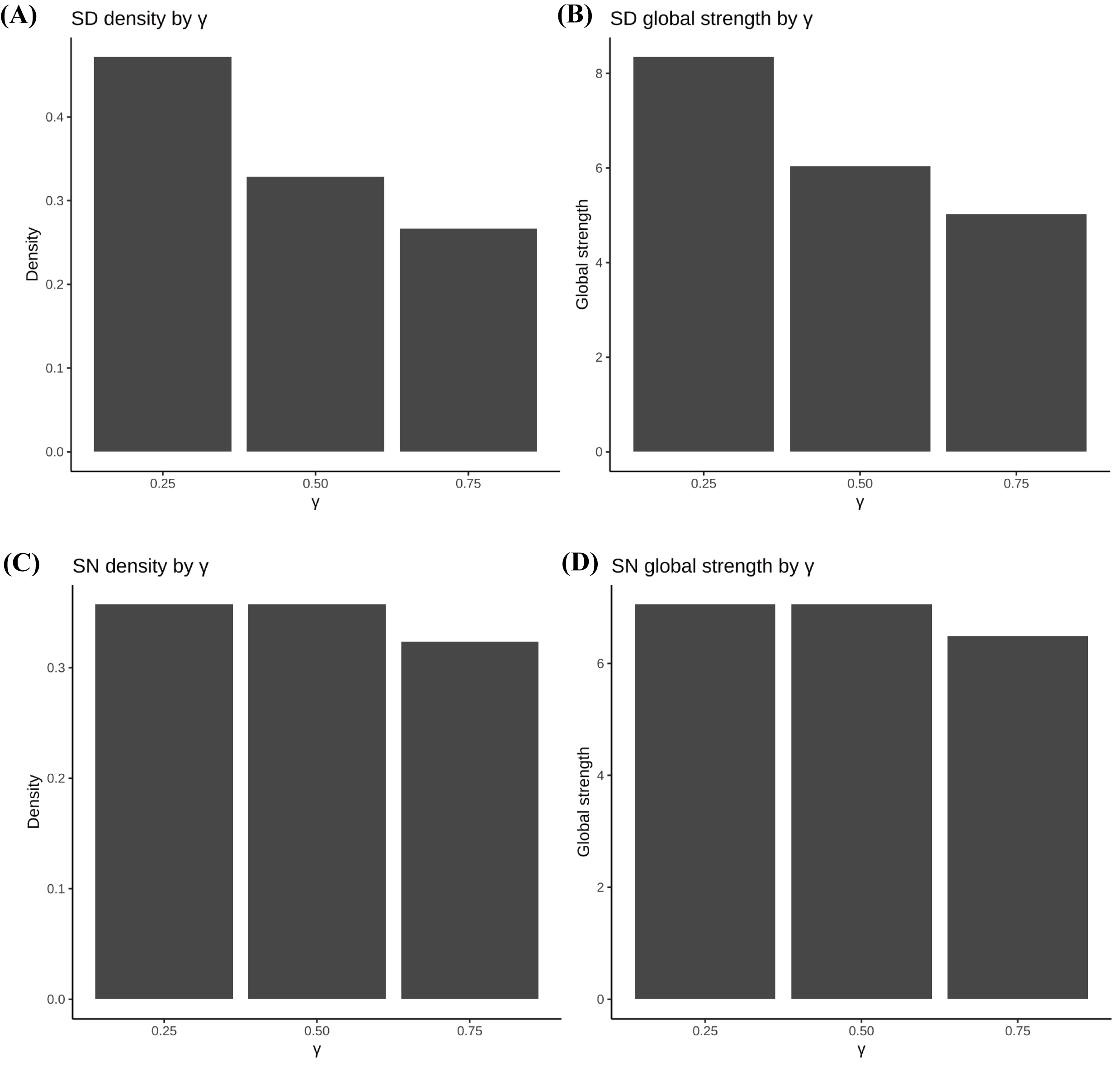

Supplement: Supplementary Table 1 — Baseline Demographic and Lifestyle Characteristics by PSQI Group (n = 570). Values are presented as median (Q1, Q3) for continuous variables and n (%) for categorical variables. P-values were calculated using Mann–Whitney U test (a), Pearson’s χ² test (b), or Fisher’s exact test (c) as appropriate. PSQI > 7 = Sleep-disturbed group; PSQI ≤ 7 = Sleep-normal group. Bold p-values indicate statistical significance at α = 0.05. [file DataSheet1.zip › Data Sheet 1/Figure S6.TIF]

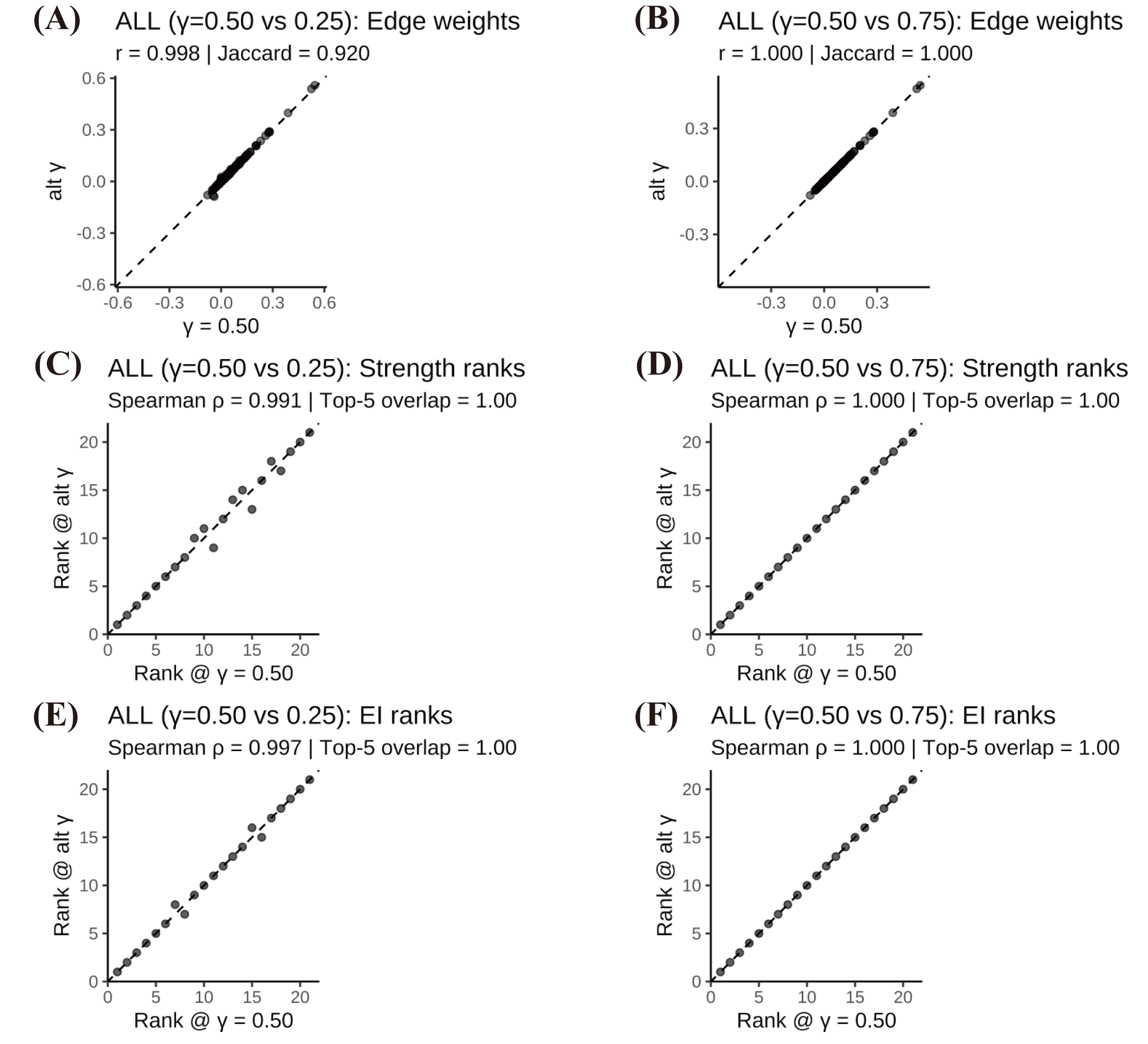

Supplement: Supplementary Table 1 — Baseline Demographic and Lifestyle Characteristics by PSQI Group (n = 570). Values are presented as median (Q1, Q3) for continuous variables and n (%) for categorical variables. P-values were calculated using Mann–Whitney U test (a), Pearson’s χ² test (b), or Fisher’s exact test (c) as appropriate. PSQI > 7 = Sleep-disturbed group; PSQI ≤ 7 = Sleep-normal group. Bold p-values indicate statistical significance at α = 0.05. [file DataSheet1.zip › Data Sheet 1/Figure S2.TIF]

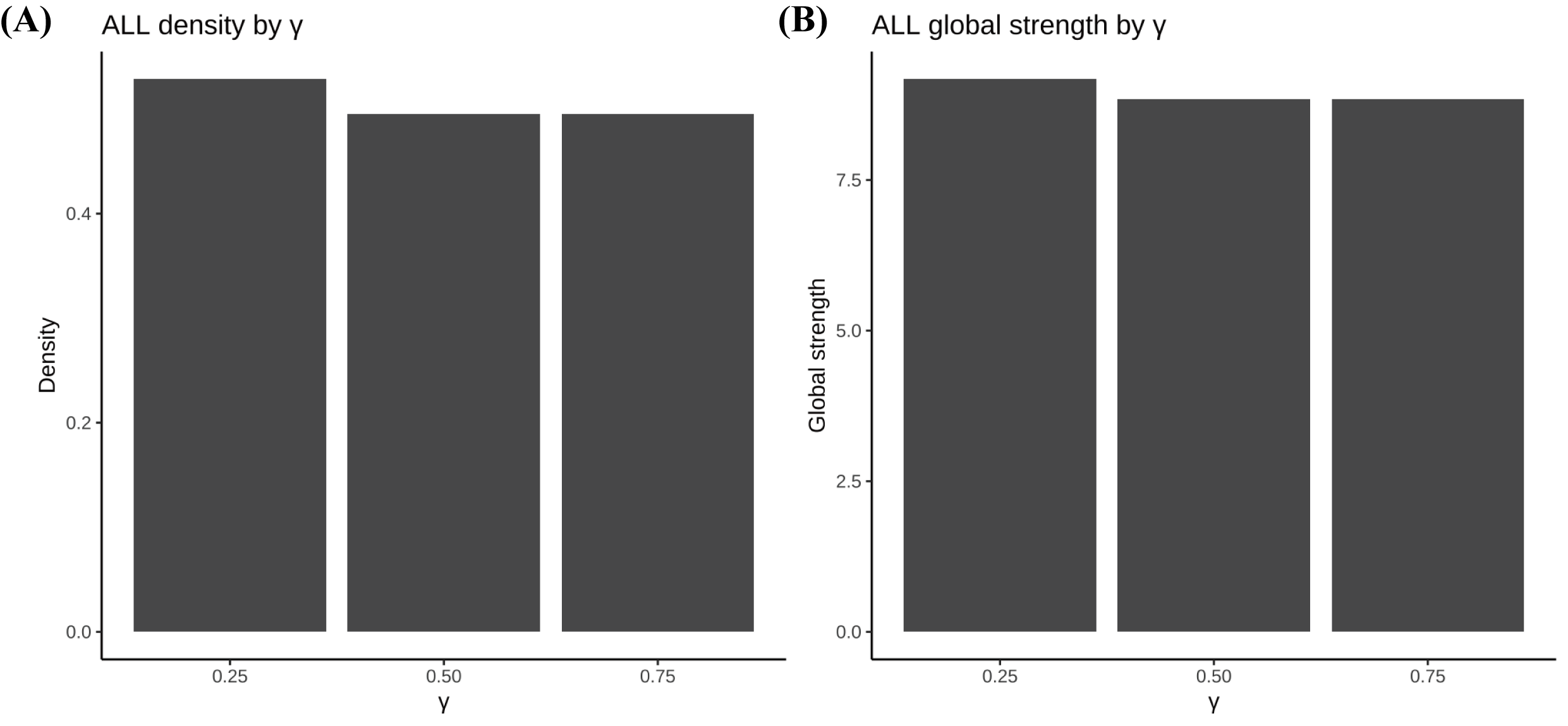

Supplement: Supplementary Table 1 — Baseline Demographic and Lifestyle Characteristics by PSQI Group (n = 570). Values are presented as median (Q1, Q3) for continuous variables and n (%) for categorical variables. P-values were calculated using Mann–Whitney U test (a), Pearson’s χ² test (b), or Fisher’s exact test (c) as appropriate. PSQI > 7 = Sleep-disturbed group; PSQI ≤ 7 = Sleep-normal group. Bold p-values indicate statistical significance at α = 0.05. [file DataSheet1.zip › Data Sheet 1/Figure S3.TIF]

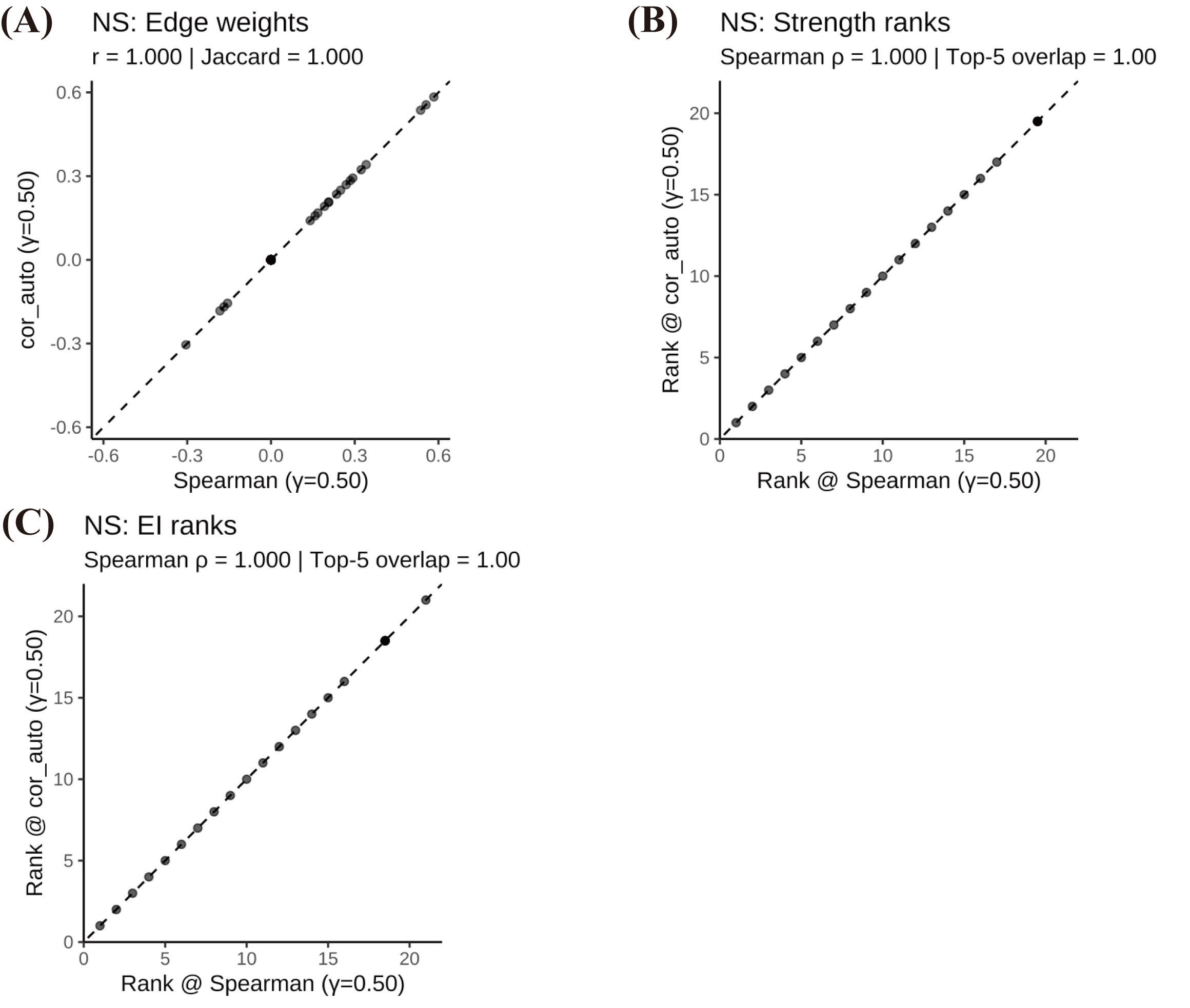

Supplement: Supplementary Table 1 — Baseline Demographic and Lifestyle Characteristics by PSQI Group (n = 570). Values are presented as median (Q1, Q3) for continuous variables and n (%) for categorical variables. P-values were calculated using Mann–Whitney U test (a), Pearson’s χ² test (b), or Fisher’s exact test (c) as appropriate. PSQI > 7 = Sleep-disturbed group; PSQI ≤ 7 = Sleep-normal group. Bold p-values indicate statistical significance at α = 0.05. [file DataSheet1.zip › Data Sheet 1/Figure S1.TIF]

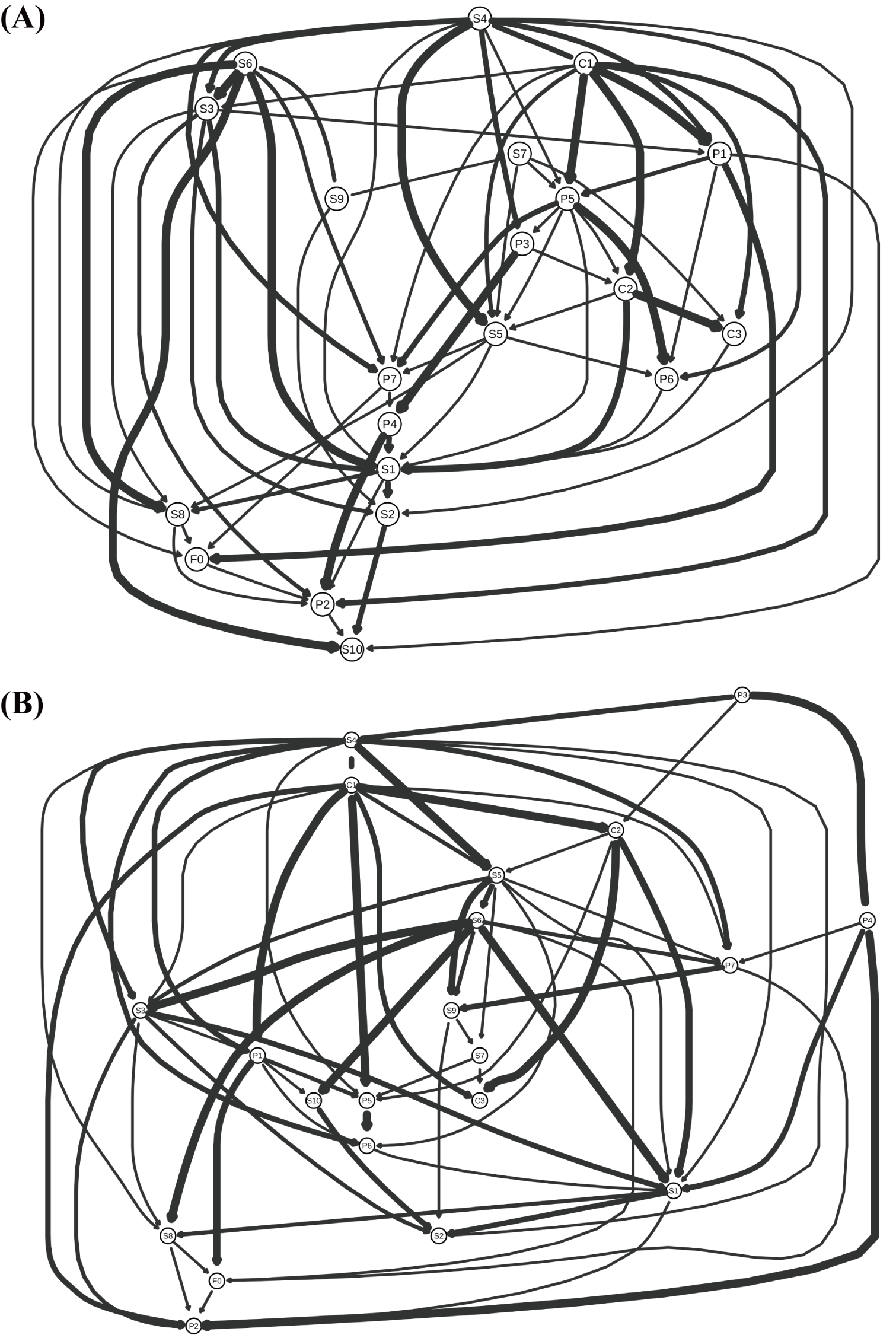

Supplement: Supplementary Table 1 — Baseline Demographic and Lifestyle Characteristics by PSQI Group (n = 570). Values are presented as median (Q1, Q3) for continuous variables and n (%) for categorical variables. P-values were calculated using Mann–Whitney U test (a), Pearson’s χ² test (b), or Fisher’s exact test (c) as appropriate. PSQI > 7 = Sleep-disturbed group; PSQI ≤ 7 = Sleep-normal group. Bold p-values indicate statistical significance at α = 0.05. [file DataSheet1.zip › Data Sheet 1/Figure S10.TIF]
