# Supplementary material for: Depression and daytime dysfunction centralize the fatigue–sleep cascade in island firefighters: a symptom network and Bayesian DAG study
Source: Front Psychiatry. 2025 Oct 29;16:1663957. doi: 10.3389/fpsyt.2025.1663957 (PMC12605024; doi:10.3389/fpsyt.2025.1663957)
Supplement: Supplementary file 2 [file Table1.docx]

Supplementary Material

## Supplementary Tables

**Supplementary Table S1.** Baseline Demographic and Lifestyle Characteristics by PSQI Group (n = 570)

| **Variable** | **Overall (*n* = 570)** | **SD (*n* = 262)** | **SN (*n* = 308)** | ***P*-value** |
| --- | --- | --- | --- | --- |
| **Age (years)** | 25.0 (23.0, 28.0) | 25.0 (24.0, 28.0) | 24.0 (23.0, 27.0) | **< 0.001^a^** |
| **Years of work (years)** | 5.0 (2.1, 8.0) | 7.0 (3.0, 9.0) | 4.5 (2.0, 8.0) | **0.003^a^** |
| **Only child, *n* (%)** |  |  |  | 0.680 ^b^ |
| Yes | 167 (29.3%) | 79 (30.2%) | 88 (28.6%) |  |
| No | 403 (70.7%) | 183 (69.8%) | 220 (71.4%) |  |
| **Marital status, *n* (%)** |  |  |  | **0.007** ^b^ |
| Unmarried | 420 (73.7%) | 179 (68.3%) | 241 (78.3%) |  |
| Married | 150 (26.3%) | 83 (31.7%) | 67 (21.8%) |  |
| **Education, *n* (%)** |  |  |  | 0.782^c^ |
| Junior college | 85 (14.9%) | 43 (16.4%) | 42 (13.6%) |  |
| Senior high school | 136 (23.9%) | 60 (22.9%) | 76 (24.7%) |  |
| Vocational college | 286 (50.2%) | 129 (49.2%) | 157 (51.0%) |  |
| Bachelor’s degree | 60 (10.5%) | 27 (10.3%) | 33 (10.7%) |  |
| Postgraduate | 3 (0.5%) | 3 (1.1%) | 0 (0.0%) |  |
| **Work schedule, *n* (%)** |  |  |  | **< 0.001^b^** |
| Non-shift work | 177 (31.1%) | 60 (22.9%) | 117 (38.0%) |  |
| Shift work | 255 (44.7%) | 144 (55.0%) | 111 (36.0%) |  |
| Irregular/on-call | 138 (24.2%) | 58 (22.1%) | 80 (26.0%) |  |
| **Smoking, *n* (%)** |  |  |  | **< 0.001^b^** |
| Yes | 362 (63.5%) | 204 (77.9%) | 158 (51.3%) |  |
| No | 208 (36.5%) | 58 (22.1%) | 150 (48.7%) |  |
| **Coffee drinking, *n* (%)** |  |  |  | **< 0.001^b^** |
| Yes | 163 (28.6%) | 122 (46.6%) | 41 (13.3%) |  |
| No | 407 (71.4%) | 140 (53.4%) | 267 (86.7%) |  |
| **Tea drinking, n (%)** |  |  |  | **< 0.001^b^** |
| Yes | 258 (45.3%) | 175 (66.8%) | 83 (26.9%) |  |
| No | 312 (54.7%) | 87 (33.2%) | 225 (73.1%) |  |
| **Use of other stimulants, *n* (%)** |  |  |  | **< 0.001^b^** |
| Yes | 230 (40.4%) | 157 (59.9%) | 73 (23.7%) |  |
| No | 340 (59.7%) | 105 (40.1%) | 235 (76.3%) |  |

**Notes.** Values are presented as median (Q1, Q3) for continuous variables and n (%) for categorical variables. P-values were calculated using Mann–Whitney U test (a), Pearson’s χ² test (b), or Fisher’s exact test (c) as appropriate. PSQI > 7 = Sleep-disturbed group; PSQI ≤ 7 = Sleep-normal group. Bold p-values indicate statistical significance at α = 0.05.

**Supplementary Table S2.** Baseline Demographic and Lifestyle Characteristics by Work Schedule (n = 432)

| **Variable** | **SW (*n* = 255)** | **NS (*n* = 177)** | ***P*-Value** |
| --- | --- | --- | --- |
| **Age (years)** | 25.0 (23.0, 28.0) | 25.0 (23.0, 28.0) | 0.275^a^ |
| **Years of work (years)** | 5.0 (3.0, 9.0) | 5.0 (3.0, 9.0) | 0.447 ^a^ |
| **Only child, *n* (%)** |  |  | 0.651^b^ |
| Yes | 63 (24.7%) | 63 (24.7%) |  |
| No | 192 (75.3%) | 192 (75.3%) |  |
| **Marital status, *n* (%)** |  |  | **0.014 ^b^** |
| Unmarried | 178 (69.8%) | 178 (69.8%) |  |
| Married | 77 (30.2%) | 77 (30.2%) |  |
| **Education, *n* (%)** |  |  | 0.138 ^c^ |
| Junior college | 34 (13.3%) | 34 (13.3%) |  |
| Senior high school | 69 (27.1%) | 69 (27.1%) |  |
| Vocational college | 124 (48.6%) | 124 (48.6%) |  |
| Bachelor’s degree | 28 (11.0%) | 28 (11.0%) |  |
| Postgraduate | 0 (0.0%) | 0 (0.0%) |  |
| **Smoking, *n* (%)** |  |  | 0.333 ^b^ |
| Yes | 167 (65.5%) | 167 (65.5%) |  |
| No | 88 (34.5%) | 88 (34.5%) |  |
| **Coffee drinking, *n* (%)** |  |  | **< 0.001 ^b^** |
| Yes | 92 (36.1%) | 92 (36.1%) |  |
| No | 163 (63.9%) | 163 (63.9%) |  |
| **Tea drinking, n (%)** |  |  | **< 0.001 ^b^** |
| Yes | 139 (54.5%) | 139 (54.5%) |  |
| No | 116 (45.5%) | 116 (45.5%) |  |
| **Use of other stimulants, *n* (%)** |  |  | **0.028 ^b^** |
| Yes | 113 (44.3%) | 113 (44.3%) |  |
| No | 142 (55.7%) | 142 (55.7%) |  |

**Notes.** Values are presented as median (Q1, Q3) for continuous variables and n (%) for categorical variables. P-values were calculated using Mann–Whitney U test (a), Pearson’s χ² test (b), or Fisher’s exact test (c) as appropriate. SW = Shift-work group; NS = Non-shift group. Bold p-values indicate statistical significance at α = 0.05.

**Supplementary Table S3.** Symptom Differences Between Sleep-Disturbed and Sleep-Normal Firefighters (n = 570)

| **Node** | **Overall (*n* = 570)** | **SD (*n* = 262)** | **SN(*n* = 308)** | ***Z*** | ***P*-value** |
| --- | --- | --- | --- | --- | --- |
| P1 | 1.00 (0.00, 2.00) | 2.00 (1.00, 2.00) | 0.00 (0.00, 1.00) | -15.583 | **<0.001** |
| P2 | 1.00 (0.00, 3.00) | 3.00 (2.00, 3.00) | 0.00 (0.00, 1.00) | -16.011 | **<0.001** |
| P3 | 1.00 (1.00, 1.00) | 1.00 (1.00, 2.00) | 1.00 (0.00, 1.00) | -11.421 | **<0.001** |
| P4 | 1.00 (0.00, 2.00) | 2.00 (1.00, 2.00) | 0.00 (0.00, 1.00) | -11.113 | **<0.001** |
| P5 | 1.00 (0.00, 1.00) | 1.00 (1.00, 2.00) | 0.00 (0.00, 1.00) | -14.719 | **<0.001** |
| P6 | 0.00 (0.00, 0.00) | 0.00 (0.00, 1.00) | 0.00 (0.00, 0.00) | -8.235 | **<0.001** |
| P7 | 1.00 (0.00, 2.00) | 2.00 (2.00, 2.00) | 0.00 (0.00, 1.00) | -18.031 | **<0.001** |
| S1 | 16.00 (14.00, 20.00) | 18.00 (16.00, 23.00) | 15.00 (13.00, 17.00) | -10.497 | **<0.001** |
| S2 | 14.00 (11.00, 17.00) | 17.00 (13.25, 21.00) | 12.00 (11.00, 14.00) | -10.399 | **<0.001** |
| S3 | 12.00 (10.00, 15.00) | 14.00 (12.00, 17.00) | 11.00 (9.00, 12.00) | -11.916 | **<0.001** |
| S4 | 18.00 (15.00, 23.00) | 22.00 (20.00, 25.00) | 15.00 (13.00, 17.00) | -15.865 | **<0.001** |
| S5 | 14.00 (11.00, 17.00) | 17.00 (14.00, 19.00) | 12.00 (11.00, 14.00) | -14.112 | **<0.001** |
| S6 | 7.00 (6.00, 10.00) | 9.00 (7.00, 11.00) | 7.00 (6.00, 8.00) | -10.216 | **<0.001** |
| S7 | 7.00 (7.00, 12.00) | 7.00 (7.00, 12.00) | 7.00 (7.00, 13.00) | 0.324 | 0.690 |
| S8 | 7.00 (6.00, 9.00) | 8.00 (6.00, 12.00) | 7.00 (6.00, 8.00) | -8.067 | **<0.001** |
| S9 | 10.00 (10.00, 16.00) | 10.00 (10.00, 17.00) | 10.00 (10.00, 12.00) | -1.034 | 0.214 |
| S10 | 9.00 (7.00, 11.00) | 11.00 (9.00, 13.00) | 8.00 (7.00, 9.00) | -13.524 | **<0.001** |
| C1 | 41.00 (36.00, 47.00) | 38.00 (33.00, 43.00) | 45.00 (39.00, 49.00) | 8.957 | **<0.001** |
| C2 | 27.00 (23.00, 30.00) | 24.00 (21.00, 28.00) | 28.00 (25.00, 31.00) | 7.540 | **<0.001** |
| C3 | 12.00 (11.00, 14.00) | 12.00 (10.00, 13.00) | 13.00 (12.00, 15.00) | 6.966 | **<0.001** |
| F0 | 23.00 (14.00, 32.00) | 32.00 (26.25, 36.75) | 14.00 (12.00, 18.25) | -17.664 | **<0.001** |

**Note.** Values are expressed as median (Q1, Q3). Group comparisons were performed using Mann–Whitney *U* tests due to non-normal distribution of most variables. SD group = Sleep-disturbed group (PSQI > 7); SN group = Sleep-normal group (PSQI ≤ 7). P = PSQI components (P1–P7); S = SCL-90 subscales (S1–S10); C = CD-RISC factors (C1–C3); F = FSS total score. All *p*-values are two-tailed; values in bold indicate statistical significance at α = 0.05.

**Supplementary Table S4.** Symptom Differences Between Shift-Work and Non-Shift Firefighters (n = 432)

| **Node** | **SW (*n* = 255)** | **NS (*n* = 177)** | ***Z*** | ***P*-value** |
| --- | --- | --- | --- | --- |
| P1 | 1.00 (1.00, 2.00) | 1.00 (0.00, 1.00) | -5.359 | **<0.001** |
| P2 | 2.00 (0.00, 3.00) | 1.00 (0.00, 2.00) | -3.524 | **<0.001** |
| P3 | 1.00 (1.00, 1.00) | 1.00 (0.00, 1.00) | -2.224 | **0.010** |
| P4 | 1.00 (0.00, 2.00) | 0.00 (0.00, 1.00) | -2.042 | **0.029** |
| P5 | 1.00 (0.00, 2.00) | 1.00 (0.00, 1.00) | -4.077 | **<0.001** |
| P6 | 0.00 (0.00, 1.00) | 0.00 (0.00, 0.00) | -1.477 | **0.046** |
| P7 | 1.00 (0.00, 2.00) | 1.00 (0.00, 2.00) | -4.131 | **<0.001** |
| S1 | 16.00 (14.00, 20.00) | 15.00 (13.00, 18.00) | -2.883 | **0.004** |
| S2 | 14.00 (11.00, 18.00) | 12.00 (11.00, 16.00) | -2.999 | **0.003** |
| S3 | 12.00 (10.00, 15.00) | 11.00 (9.00, 14.00) | -3.477 | **<0.001** |
| S4 | 20.00 (15.00, 24.00) | 16.00 (14.00, 21.00) | -4.258 | **<0.001** |
| S5 | 15.00 (12.00, 17.00) | 13.00 (11.00, 16.00) | -2.933 | **0.003** |
| S6 | 8.00 (6.00, 10.00) | 7.00 (6.00, 9.00) | -1.968 | **0.044** |
| S7 | 7.00 (7.00, 12.00) | 7.00 (7.00, 12.00) | 0.163 | 0.840 |
| S8 | 8.00 (6.00, 9.00) | 7.00 (6.00, 8.00) | -3.182 | **0.001** |
| S9 | 10.00 (10.00, 16.00) | 10.00 (10.00, 16.00) | -0.768 | 0.354 |
| S10 | 9.00 (8.00, 12.00) | 9.00 (7.00, 10.00) | -3.796 | **<0.001** |
| C1 | 41.00 (36.00, 46.00) | 43.00 (37.00, 49.00) | 2.272 | **0.023** |
| C2 | 26.00 (23.00, 29.00) | 28.00 (24.00, 30.00) | 2.593 | **0.009** |
| C3 | 12.00 (11.00, 14.00) | 13.00 (11.00, 15.00) | 1.894 | 0.057 |
| F0 | 26.00 (15.50, 33.00) | 16.00 (12.00, 28.00) | -4.998 | **<0.001** |

**Note.** Values are expressed as median (Q1, Q3). Group comparisons were performed using Mann–Whitney *U* tests due to non-normal distribution of most variables. SD = Sleep-disturbed group (PSQI > 7); SN = Sleep-normal group (PSQI ≤ 7); P = PSQI components (P1–P7); S = SCL-90 subscales (S1–S10); C = CD-RISC factors (C1–C3); F = FSS total score. All *p*-values are two-tailed; values in bold indicate statistical significance at α = 0.05.

**Supplementary Table S5.** Robustness of Symptom Networks to Correlation Estimators (Spearman vs cor_auto; γ = 0.50)

| Ref. Comparison | Overall (γ=0.50) | | SD (γ=0.50) | | SN (γ=0.50) | | SW (γ=0.50) | | NS (γ=0.50) | |
| --- | --- | --- | --- | --- | --- | --- | --- | --- | --- | --- |
|  | Spearman (Ref) | cor_auto (Alt) | Spearman (Ref) | cor_auto (Alt) | Spearman (Ref) | cor_auto (Alt) | Spearman (Ref) | cor_auto (Alt) | Spearman (Ref) | cor_auto (Alt) |
| Jaccard | 1.000 | 1.000 | 1.000 | 1.000 | 1.000 | 1.000 | 1.000 | 1.000 | 1.000 | 1.000 |
| Edge r | 1.000 | 1.000 | 1.000 | 1.000 | 1.000 | 1.000 | 1.000 | 1.000 | 1.000 | 1.000 |
| Sign Agreement | 1.000 | 1.000 | 1.000 | 1.000 | 1.000 | 1.000 | 1.000 | 1.000 | 1.000 | 1.000 |
| ρ (Strength) | 1.000 | 1.000 | 1.000 | 1.000 | 1.000 | 1.000 | 1.000 | 1.000 | 1.000 | 1.000 |
| ρ (EI) | 1.000 | 1.000 | 1.000 | 1.000 | 1.000 | 1.000 | 1.000 | 1.000 | 1.000 | 1.000 |
| Density | 0.176 | 0.176 | 0.057 | 0.057 | 0.062 | 0.062 | 0.095 | 0.095 | 0.095 | 0.095 |
| GStrength | 7.087 | 7.087 | 3.526 | 3.526 | 4.034 | 4.034 | 4.981 | 4.981 | 5.554 | 5.554 |
| Top-3 Strength | 1.000 | 1.000 | 1.000 | 1.000 | 1.000 | 1.000 | 1.000 | 1.000 | 1.000 | 1.000 |
| Top-5 Strength | 1.000 | 1.000 | 1.000 | 1.000 | 1.000 | 1.000 | 1.000 | 1.000 | 1.000 | 1.000 |
| Top-3 EI | 1.000 | 1.000 | 1.000 | 1.000 | 1.000 | 1.000 | 1.000 | 1.000 | 1.000 | 1.000 |
| Top-5 EI | 1.000 | 1.000 | 1.000 | 1.000 | 1.000 | 1.000 | 1.000 | 1.000 | 1.000 | 1.000 |

**Notes.** ALL = full sample; SN = sleep-normal; SD = sleep-disturbed; NS = non-shift; SW = shift-work; EI = Expected Influence. Networks were estimated with EBICglasso at γ = 0.50. “Spearman” uses rank correlations; “cor_auto” uses polychoric/tetrachoric correlations for ordinal items. Density = proportion of non-zero edges; Global strength = sum of absolute edge weights; Top-k overlap = proportion of top-k nodes (by Strength) preserved across estimators.

**Supplementary Table S6.** Structural and Centrality Stability of Symptom Networks Under Varying EBIC Hyperparameters (γ = 0.25, 0.50, 0.75)

| Ref. Comparison | Overall(γ=0.50) | | SD (γ=0.50) | | SN (γ=0.50) | | SW (γ=0.50) | | NS (γ=0.50) | |
| --- | --- | --- | --- | --- | --- | --- | --- | --- | --- | --- |
|  | γ=0.25 | γ=0.75 | γ=0.25 | γ=0.75 | γ=0.25 | γ=0.75 | γ=0.25 | γ=0.75 | γ=0.25 | γ=0.75 |
| Jaccard | 0.920 | 1.000 | 0.647 | 0.812 | 1.000 | 0.907 | 1.000 | 1.000 | 0.863 | 1.000 |
| Edge r | 0.998 | 1.000 | 0.976 | 0.993 | 1.000 | 0.998 | 1.000 | 1.000 | 0.991 | 1.000 |
| Sign Agreement | 1.000 | 1.000 | 1.000 | 1.000 | 1.000 | 1.000 | 1.000 | 1.000 | 1.000 | 1.000 |
| ρ (Strength) | 0.991 | 1.000 | 0.970 | 0.990 | 1.000 | 0.998 | 1.000 | 1.000 | 0.973 | 1.000 |
| ρ (EI) | 0.997 | 1.000 | 0.966 | 0.988 | 1.000 | 0.997 | 1.000 | 1.000 | 0.986 | 1.000 |
| Density (Ref γ) | 0.495 | 0.495 | 0.329 | 0.329 | 0.357 | 0.357 | 0.452 | 0.452 | 0.429 | 0.429 |
| Density (Alt γ) | 0.529 | 0.495 | 0.471 | 0.267 | 0.357 | 0.324 | 0.452 | 0.452 | 0.476 | 0.429 |
| GStrength (Ref γ) | 8.848 | 8.848 | 6.041 | 6.041 | 7.053 | 7.053 | 8.465 | 8.465 | 8.343 | 8.343 |
| GStrength (Alt γ) | 9.174 | 8.848 | 8.345 | 5.021 | 7.053 | 6.491 | 8.465 | 8.465 | 9.153 | 8.343 |
| Top-3 Strength | 1.000 | 1.000 | 1.000 | 0.667 | 1.000 | 1.000 | 1.000 | 1.000 | 0.667 | 1.000 |
| Top-5 Strength | 1.000 | 1.000 | 1.000 | 1.000 | 1.000 | 1.000 | 1.000 | 1.000 | 1.000 | 1.000 |
| Top-10 Strength | 0.900 | 1.000 | 0.900 | 0.900 | 1.000 | 1.000 | 1.000 | 1.000 | 0.900 | 1.000 |
| Top-3 EI | 1.000 | 1.000 | 1.000 | 1.000 | 1.000 | 1.000 | 1.000 | 1.000 | 1.000 | 1.000 |
| Top-5 EI | 1.000 | 1.000 | 0.800 | 0.800 | 1.000 | 1.000 | 1.000 | 1.000 | 1.000 | 1.000 |
| Top-10 EI | 1.000 | 1.000 | 0.800 | 1.000 | 1.000 | 1.000 | 1.000 | 1.000 | 0.900 | 1.000 |

**Notes.** This table summarizes the structural and centrality stability metrics of the estimated symptom networks under varying EBIC hyperparameter values (γ = 0.25, 0.50, 0.75). The reference network is estimated using γ = 0.50, while γ = 0.25 and γ = 0.75 serve as sensitivity comparisons. SD = sleep-disturbed; SN = sleep-normal; SW = shift-work; NS = non-shift; EI = Expected Influence; Jaccard = Jaccard similarity index of edge presence; Edge r = Pearson correlation of edge weights; Sign Agreement = proportion of consistent edge signs; ρ (Strength) and ρ (EI) = Spearman correlations of node-level strength and expected influence; Density = proportion of nonzero edges; GStrength = Global Strength (sum of absolute edge weights); Top-k = proportion of top-k ranked nodes (k = 3, 5, 10) preserved across networks.

**Supplementary Table S7.** Top 5 Strongest Edges and 95% Confidence Intervals in Each Subgroup Network

|  | **Node1** | **Node2** | **Weight** | **CI-low** | **CI-high** |
| --- | --- | --- | --- | --- | --- |
| Overall |  |  |  |  |  |
|  | C1 | C2 | 0.545 | 0.482 | 0.600 |
|  | P3 | P4 | 0.525 | 0.469 | 0.575 |
|  | C2 | C3 | 0.389 | 0.323 | 0.450 |
|  | P7 | F0 | 0.282 | 0.212 | 0.342 |
|  | P5 | P7 | 0.281 | 0.210 | 0.340 |
| SD |  |  |  |  |  |
|  | C1 | C2 | 0.413 | 0.342 | 0.520 |
|  | P3 | P4 | 0.410 | 0.344 | 0.527 |
|  | C2 | C3 | 0.329 | 0.257 | 0.434 |
|  | P5 | P6 | 0.284 | 0.188 | 0.419 |
|  | S4 | S5 | 0.233 | 0.140 | 0.322 |
| SN |  |  |  |  |  |
|  | C1 | C2 | 0.499 | 0.394 | 0.550 |
|  | P3 | P4 | 0.472 | 0.371 | 0.515 |
|  | P1 | P5 | 0.374 | 0.253 | 0.451 |
|  | C2 | C3 | 0.358 | 0.254 | 0.418 |
|  | P5 | P7 | 0.342 | 0.231 | 0.413 |
| SW |  |  |  |  |  |
|  | C1 | C2 | 0.479 | 0.391 | 0.559 |
|  | P3 | P4 | 0.436 | 0.336 | 0.511 |
|  | C2 | C3 | 0.373 | 0.275 | 0.463 |
|  | S4 | S5 | 0.305 | 0.216 | 0.389 |
|  | P7 | F0 | 0.297 | 0.205 | 0.385 |
| NS |  |  |  |  |  |
|  | C1 | C2 | 0.473 | 0.363 | 0.580 |
|  | P3 | P4 | 0.449 | 0.344 | 0.542 |
|  | P1 | P5 | 0.442 | 0.335 | 0.561 |
|  | C2 | C3 | 0.307 | 0.189 | 0.410 |
|  | P5 | P7 | 0.270 | 0.165 | 0.376 |

**Notes.** This table lists the top 5 edges with the highest absolute weights in each estimated network (overall and subgroups). The edge weight reflects the strength of association between nodes. 95% nonparametric bootstrap confidence intervals were computed for each edge. SD = sleep-disturbed group; SN = sleep-normal group; SW = shift-work group; NS = non-shift group.

**Supplementary Table S8.** Comparison of Edge Strength and Directional Consistency Between Tabu and HC Algorithms (γ = 0.50)

| Pair | Tabu | | | | HC | | | agree |
| --- | --- | --- | --- | --- | --- | --- | --- | --- |
|  | Arc | Strength | Direction | Arc | | Strength | Direction |  |
| C1--P2 | C1->P2 | 0.800 | 0.915 | C1->P2 | | 0.803 | 0.915 | TRUE |
| P2--S3 | S3->P2 | 0.525 | 0.883 | S3->P2 | | 0.536 | 0.880 | TRUE |
| S10--S6 | S6->S10 | 0.947 | 0.862 | S6->S10 | | 0.951 | 0.857 | TRUE |
| S5--S9 | S5->S9 | 0.907 | 0.857 | S5->S9 | | 0.903 | 0.852 | TRUE |
| P7--S4 | S4->P7 | 0.625 | 0.843 | S4->P7 | | 0.632 | 0.844 | TRUE |
| P2--P4 | P4->P2 | 1.000 | 0.831 | P4->P2 | | 0.999 | 0.840 | TRUE |
| F0--P1 | P1->F0 | 0.872 | 0.831 | P1->F0 | | 0.874 | 0.838 | TRUE |
| S6--S8 | S6->S8 | 0.976 | 0.840 | S6->S8 | | 0.980 | 0.830 | TRUE |
| C1--P1 | C1->P1 | 0.972 | 0.794 | C1->P1 | | 0.971 | 0.791 | TRUE |
| P5--P7 | P5->P7 | 0.803 | 0.747 | P5->P7 | | 0.791 | 0.747 | TRUE |
| S2--S3 | S3->S2 | 0.580 | 0.734 | S3->S2 | | 0.598 | 0.731 | TRUE |
| C1--C3 | C1->C3 | 0.817 | 0.737 | C1->C3 | | 0.822 | 0.701 | TRUE |
| S1--S2 | S1->S2 | 0.782 | 0.713 | S1->S2 | | 0.780 | 0.698 | TRUE |
| C1--P5 | C1->P5 | 0.941 | 0.691 | C1->P5 | | 0.950 | 0.697 | TRUE |
| S3--S4 | S4->S3 | 0.731 | 0.688 | S4->S3 | | 0.726 | 0.684 | TRUE |
| C2--S1 | C2->S1 | 0.904 | 0.635 | C2->S1 | | 0.896 | 0.647 | TRUE |
| S1--S8 | S1->S8 | 0.544 | 0.648 | S1->S8 | | 0.543 | 0.639 | TRUE |
| P5--P6 | P5->P6 | 1.000 | 0.653 | P5->P6 | | 1.000 | 0.636 | TRUE |
| S1--S3 | S3->S1 | 0.735 | 0.630 | S3->S1 | | 0.734 | 0.629 | TRUE |
| S5--S6 | S5->S6 | 0.703 | 0.624 | S5->S6 | | 0.701 | 0.619 | TRUE |
| C2--C3 | C2->C3 | 1.000 | 0.620 | C2->C3 | | 1.000 | 0.617 | TRUE |
| C1--S4 | C1->S4 | 0.788 | 0.599 | C1->S4 | | 0.795 | 0.612 | TRUE |
| P3--P4 | P3->P4 | 1.000 | 0.636 | P3->P4 | | 1.000 | 0.612 | TRUE |
| S3--S5 | S5->S3 | 0.538 | 0.618 | S5->S3 | | 0.541 | 0.602 | TRUE |
| S1--S6 | S6->S1 | 0.921 | 0.586 | S6->S1 | | 0.927 | 0.595 | TRUE |
| C1--C2 | C1->C2 | 1.000 | 0.632 | C1->C2 | | 1.000 | 0.595 | TRUE |
| P1--S4 | S4->P1 | 0.632 | 0.565 | S4->P1 | | 0.630 | 0.568 | TRUE |
| P3--S5 | S5->P3 | 0.859 | 0.558 | S5->P3 | | 0.855 | 0.554 | TRUE |
| S3--S6 | S6->S3 | 0.986 | 0.534 | S6->S3 | | 0.987 | 0.551 | TRUE |
| S4--S5 | S4->S5 | 0.987 | 0.551 | S4->S5 | | 0.989 | 0.541 | TRUE |
| P4--S1 | P4->S1 | 0.669 | 0.509 | P4->S1 | | 0.659 | 0.530 | TRUE |
| P6--S4 | S4->P6 | 0.599 | 0.534 | S4->P6 | | 0.602 | 0.528 | TRUE |
| P7--S9 | P7->S9 | 0.652 | 0.522 | P7->S9 | | 0.632 | 0.523 | TRUE |
| P3--S4 | S4->P3 | 0.818 | 0.533 | S4->P3 | | 0.828 | 0.522 | TRUE |
| S10--S2 | S2->S10 | 0.706 | 0.511 | S10->S2 | | 0.700 | 0.502 | FALSE |

**Notes.** This table compares the edge strength and directional probabilities of arcs identified by the Tabu and Hill-Climbing (HC) algorithms in the estimated Bayesian DAG (Directed Acyclic Graph) models (bootstrap repetitions R = 5000). “Arc” denotes the estimated directed edge between two nodes. “Strength” represents the bootstrap frequency of arc presence, and “Direction” denotes the estimated directional probability of each arc. “Agreement” indicates whether both algorithms identified the same arc with consistent direction. Only arcs with bootstrap presence frequency ≥ 0.50 are included.

**Supplementary Table S9.** Comparison of Edge Strength and Directional Consistency Between Tabu and HC Algorithms (γ = 0.20)

| Pair | Tabu | | | HC | | | agree |
| --- | --- | --- | --- | --- | --- | --- | --- |
|  | Arc | Strength | Direction | Arc | Strength | Direction |  |
| C1--P2 | C1->P2 | 0.800 | 0.915 | C1->P2 | 0.803 | 0.915 | TRUE |
| P2--S1 | S1->P2 | 0.330 | 0.894 | S1->P2 | 0.328 | 0.905 | TRUE |
| P2--S3 | S3->P2 | 0.525 | 0.883 | S3->P2 | 0.536 | 0.880 | TRUE |
| S10--S6 | S6->S10 | 0.947 | 0.862 | S6->S10 | 0.951 | 0.857 | TRUE |
| S5--S9 | S5->S9 | 0.907 | 0.857 | S5->S9 | 0.903 | 0.852 | TRUE |
| P2--S8 | S8->P2 | 0.211 | 0.854 | S8->P2 | 0.208 | 0.845 | TRUE |
| F0--S8 | S8->F0 | 0.361 | 0.853 | S8->F0 | 0.346 | 0.860 | TRUE |
| P7--S4 | S4->P7 | 0.625 | 0.843 | S4->P7 | 0.632 | 0.844 | TRUE |
| S6--S8 | S6->S8 | 0.976 | 0.840 | S6->S8 | 0.980 | 0.830 | TRUE |
| C1--P7 | C1->P7 | 0.352 | 0.835 | C1->P7 | 0.349 | 0.827 | TRUE |
| F0--P1 | P1->F0 | 0.872 | 0.831 | P1->F0 | 0.874 | 0.838 | TRUE |
| P2--P4 | P4->P2 | 1.000 | 0.831 | P4->P2 | 0.999 | 0.840 | TRUE |
| P7--S5 | S5->P7 | 0.372 | 0.819 | S5->P7 | 0.384 | 0.818 | TRUE |
| C1--P1 | C1->P1 | 0.972 | 0.794 | C1->P1 | 0.971 | 0.791 | TRUE |
| P1--S10 | P1->S10 | 0.217 | 0.777 | P1->S10 | 0.212 | 0.767 | TRUE |
| P5--P7 | P5->P7 | 0.803 | 0.747 | P5->P7 | 0.791 | 0.747 | TRUE |
| S3--S8 | S3->S8 | 0.267 | 0.739 | S3->S8 | 0.260 | 0.711 | TRUE |
| C1--C3 | C1->C3 | 0.817 | 0.737 | C1->C3 | 0.822 | 0.701 | TRUE |
| S2--S3 | S3->S2 | 0.580 | 0.734 | S3->S2 | 0.598 | 0.731 | TRUE |
| S2--S4 | S4->S2 | 0.305 | 0.725 | S4->S2 | 0.305 | 0.705 | TRUE |
| F0--P7 | P7->F0 | 0.258 | 0.720 | P7->F0 | 0.275 | 0.730 | TRUE |
| C2--S5 | C2->S5 | 0.325 | 0.718 | C2->S5 | 0.333 | 0.703 | TRUE |
| S1--S2 | S1->S2 | 0.782 | 0.713 | S1->S2 | 0.780 | 0.698 | TRUE |
| F0--P2 | F0->P2 | 0.436 | 0.705 | F0->P2 | 0.423 | 0.717 | TRUE |
| F0--S6 | S6->F0 | 0.219 | 0.694 | S6->F0 | 0.222 | 0.671 | TRUE |
| C1--P5 | C1->P5 | 0.941 | 0.691 | C1->P5 | 0.950 | 0.697 | TRUE |
| S6--S9 | S6->S9 | 0.482 | 0.689 | S6->S9 | 0.476 | 0.680 | TRUE |
| S3--S4 | S4->S3 | 0.731 | 0.688 | S4->S3 | 0.726 | 0.684 | TRUE |
| S4--S8 | S4->S8 | 0.249 | 0.684 | S4->S8 | 0.246 | 0.670 | TRUE |
| P3--P5 | P5->P3 | 0.418 | 0.683 | P5->P3 | 0.413 | 0.652 | TRUE |
| C2--P3 | P3->C2 | 0.413 | 0.655 | P3->C2 | 0.411 | 0.628 | TRUE |
| P5--P6 | P5->P6 | 1.000 | 0.653 | P5->P6 | 1.000 | 0.636 | TRUE |
| S1--S8 | S1->S8 | 0.544 | 0.648 | S1->S8 | 0.543 | 0.639 | TRUE |
| S1--S4 | S4->S1 | 0.296 | 0.648 | S4->S1 | 0.292 | 0.640 | TRUE |
| S7--S9 | S9->S7 | 0.345 | 0.647 | S9->S7 | 0.338 | 0.651 | TRUE |
| C1--S5 | C1->S5 | 0.481 | 0.641 | C1->S5 | 0.473 | 0.653 | TRUE |
| P7--S6 | S6->P7 | 0.507 | 0.636 | S6->P7 | 0.494 | 0.648 | TRUE |
| P3--P4 | P3->P4 | 1.000 | 0.636 | P3->P4 | 1.000 | 0.612 | TRUE |
| C2--S1 | C2->S1 | 0.904 | 0.635 | C2->S1 | 0.896 | 0.647 | TRUE |
| C1--C2 | C1->C2 | 1.000 | 0.632 | C1->C2 | 1.000 | 0.595 | TRUE |
| S1--S3 | S3->S1 | 0.735 | 0.630 | S3->S1 | 0.734 | 0.629 | TRUE |
| S5--S6 | S5->S6 | 0.703 | 0.624 | S5->S6 | 0.701 | 0.619 | TRUE |
| P6--S1 | P6->S1 | 0.246 | 0.621 | P6->S1 | 0.233 | 0.634 | TRUE |
| C2--C3 | C2->C3 | 1.000 | 0.620 | C2->C3 | 1.000 | 0.617 | TRUE |
| S3--S5 | S5->S3 | 0.538 | 0.618 | S5->S3 | 0.541 | 0.602 | TRUE |
| S1--S5 | S5->S1 | 0.361 | 0.613 | S5->S1 | 0.376 | 0.613 | TRUE |
| C1--S4 | C1->S4 | 0.788 | 0.599 | C1->S4 | 0.795 | 0.612 | TRUE |
| P5--S5 | P5->S5 | 0.273 | 0.595 | P5->S5 | 0.270 | 0.573 | TRUE |
| P6--S5 | S5->P6 | 0.389 | 0.590 | S5->P6 | 0.401 | 0.605 | TRUE |
| S1--S6 | S6->S1 | 0.921 | 0.586 | S6->S1 | 0.927 | 0.595 | TRUE |
| P1--P5 | P1->P5 | 0.490 | 0.580 | P1->P5 | 0.493 | 0.585 | TRUE |
| P1--P6 | P1->P6 | 0.261 | 0.577 | P1->P6 | 0.245 | 0.611 | TRUE |
| P1--S4 | S4->P1 | 0.632 | 0.565 | S4->P1 | 0.630 | 0.568 | TRUE |
| C3--S7 | S7->C3 | 0.205 | 0.562 | S7->C3 | 0.215 | 0.569 | TRUE |
| P3--S5 | S5->P3 | 0.859 | 0.558 | S5->P3 | 0.855 | 0.554 | TRUE |
| S4--S5 | S4->S5 | 0.987 | 0.551 | S4->S5 | 0.989 | 0.541 | TRUE |
| P5--S7 | S7->P5 | 0.230 | 0.548 | S7->P5 | 0.217 | 0.511 | TRUE |
| P5--S9 | P5->S9 | 0.366 | 0.547 | P5->S9 | 0.375 | 0.554 | TRUE |
| S2--S9 | S9->S2 | 0.241 | 0.537 | S9->S2 | 0.248 | 0.530 | TRUE |
| P6--S4 | S4->P6 | 0.599 | 0.534 | S4->P6 | 0.602 | 0.528 | TRUE |
| S3--S6 | S6->S3 | 0.986 | 0.534 | S6->S3 | 0.987 | 0.551 | TRUE |
| P3--S4 | S4->P3 | 0.818 | 0.533 | S4->P3 | 0.828 | 0.522 | TRUE |
| P7--S9 | P7->S9 | 0.652 | 0.522 | P7->S9 | 0.632 | 0.523 | TRUE |
| C2--P5 | P5->C2 | 0.222 | 0.517 | C2->P5 | 0.208 | 0.520 | FALSE |
| C1--S3 | C1->S3 | 0.219 | 0.517 | C1->S3 | 0.216 | 0.515 | TRUE |
| P5--S4 | S4->P5 | 0.329 | 0.514 | S4->P5 | 0.328 | 0.519 | TRUE |
| S10--S2 | S2->S10 | 0.706 | 0.511 | S10->S2 | 0.700 | 0.502 | FALSE |
| P4--P7 | P7->P4 | 0.249 | 0.510 | P4->P7 | 0.260 | 0.513 | FALSE |
| P4--S1 | P4->S1 | 0.669 | 0.509 | P4->S1 | 0.659 | 0.530 | TRUE |
| S5--S7 | S7->S5 | 0.259 | 0.504 | S5->S7 | 0.251 | 0.533 | FALSE |
| P1--S3 | P1->S3 | 0.420 | 0.500 | S3->P1 | 0.410 | 0.511 | FALSE |

**Notes.** This table compares the edge strength and directional probabilities of arcs identified by the Tabu and Hill-Climbing (HC) algorithms in the estimated Bayesian DAG (Directed Acyclic Graph) models (bootstrap repetitions R = 5000). “Arc” denotes the estimated directed edge between two nodes. “Strength” represents the bootstrap frequency of arc presence, and “Direction” denotes the estimated directional probability of each arc. “Agreement” indicates whether both algorithms identified the same arc with consistent direction. Only arcs with bootstrap presence frequency ≥ 0.50 are included.

**Supplementary Table 10.** Number of Arcs Retained under Different Bootstrap Thresholds and Algorithms in the Sleep Disturbance Subgroup (n=262)

| Algorithm | Bootstrap Threshold | Edges Retained (pairs_at_thr) | Directed Arcs in Averaged DAG |
| --- | --- | --- | --- |
| Tabu | 0.5 | 36 | 36 |
| Tabu | 0.2 | 75 | 70 |
| Hill-Climbing | 0.5 | 35 | 35 |
| Hill-Climbing | 0.2 | 71 | 66 |

Note. This table summarizes the number of node pairs (edges) and directed arcs retained in the averaged DAGs, generated using 5,000 bootstrap samples. Two structure learning algorithms (Tabu and Hill-Climbing) were applied, and two arc frequency thresholds were tested (≥0.50 and ≥0.20). "Edges Retained" refers to the number of node pairs with arc frequency exceeding the specified threshold; "Directed Arcs" refers to the number of directed edges retained in the final averaged DAG under each condition.
